# Elucidating the mechanism of soybean-derived protein hydrolysate in stabilizing rice yield and enhancing agronomic efficiency

**DOI:** 10.3389/fpls.2025.1651406

**Published:** 2025-10-22

**Authors:** Shunchang Zhang, Lijuan Tang, Xing Zhan, Dianwen Wang, Anning Zhang, Hao Wu, Cheng Huang, Hongping Chen, Jilin Wang

**Affiliations:** ^1^ Rice National Engineering Research Center (Nanchang), Rice Research Institute, Jiangxi Academy of Agricultural Sciences, Nanchang, Jiangxi, China; ^2^ Zhejiang Taizhou Agricultural Materials Co., Ltd., Taizhou, Zhejiang, China; ^3^ Qiteng Agricultural Technology (Shenzhen) Co., Ltd., Shenzhen, Guangzhou, China; ^4^ Key Laboratory of Grain Crop Genetic Resources Evaluation and Utilization, Ministry of Agriculture and Rural Affairs, Shanghai Agrobiological Gene Center, Shanghai, China

**Keywords:** rice, protein hydrolysates, yield improvement, stress tolerance, transcriptome

## Abstract

**Introduction:**

As a type of biostimulant, protein hydrolysates (PHs) can promote crop growth, increase yield, and enhance crop tolerance to abiotic stresses. However, their application and research in rice production remain relatively limited.

**Methods:**

Focusing on“Lifenggu” (a soybean-derived protein hydrolysate), this study carried out multilocation field trials to evaluate the real-world application efficacy of this biostimulant on rice production across varying environmental conditions. Meanwhile, laboratory-based assays were conducted to analyze the doseresponse of rice growth to “Lifenggu” and its protective mechanisms under high-temperature and herbicide stress.

**Results:**

Field experimental results showed that “Lifenggu” could increase rice yield by 8.9%-14% (with an average increase of 10%). Physiological analysis revealed that “Lifenggu” might promote biomass accumulation by increasing the SPAD value of rice and enhancing the activity of nitrogen metabolic enzymes. Under herbicide and high-temperature stress, “Lifenggu” could alleviate the adverse effects caused by stress and reduce yield losses, possibly by increasing the activity of antioxidant enzymes and the content of proline, while decreasing the contents of malondialdehyde (MDA) and hydrogen peroxide (H2O2). Further transcriptomic analyses demonstrated that “Lifenggu” regulates the expression of genes involved in phytohormone biosynthesis, stress response pathways, and secondary metabolism.

**Discussion:**

This, in turn, serves as the molecular mechanism enabling its dual functions of promoting rice growth and improving stress tolerance. These results deepen insights into the yield-increasing effects of protein hydrolysates in rice, and offer both theoretical support and practical recommendations for their application in rice cultivation.

## 1 Introduction

Amidst global climate change and escalating population pressures, agricultural systems face the urgent challenge of sustaining yield growth while mitigating the impacts of environmental stressors (Searchinger, 2013). In this context, the development of safe and effective phytoregulators and abiotic stress mitigators has emerged as a critical focus in modern agronomic research. Crop production worldwide is increasingly threatened by multiple abiotic stresses, including high temperature, drought, and herbicide-induced phytotoxicity, which collectively reduce yields and pose significant risks to global food security (Carillo et al., 2025). As a staple food crop for over half of the global population, rice (*Oryza sativa* L) productivity and quality are directly linked to food security (Qiao et al., 2021). In recent years, protein hydrolysates (PHs), a class of natural-derived biostimulants, have garnered substantial attention in rice research due to their dual potential to enhance crop growth and stress resilience. Unlike traditional chemical fertilizers, PHs modulate plant physiology by promoting nutrient uptake, activating antioxidant defenses, and regulating gene expression, these properties that make them promising tools for sustainable agriculture under changing climatic conditions.

Protein hydrolysates (PHs) are mixtures of peptides and amino acids generated by chemical or enzymatic hydrolysis of proteins into lower molecular weight fractions (Schaafsma, 2009), systematically classified into animal-derived, plant-derived, and microbial-derived categories based on their origin (Du Jardin, 2015). As critical bioactive mediators in plant growth regulation, PHs exert multifaceted effects on developmental processes by directly participating in plant metabolic pathways through the provision of bioactive peptides and amino acids, thereby promoting cell division, elongation, and influencing overall growth and development (Colla et al., 2017b; van Oosten et al., 2017). They enhance nutrient uptake (e.g., potassium, magnesium), stimulate primary and secondary metabolite biosynthesis, and improve stress resistance and crop yield (Rouphael et al., 2018; Leporino et al., 2024). For instance, Leporino et al. (2024) revealed through metabolomic analysis that PHs regulate dipeptide, fatty acid, and phenolic compound levels in tomato plants, coordinating primary and secondary metabolism, while Rouphael et al. (2017) showed that foliar application of legume-based PHs enhanced potassium and magnesium absorption, photosynthetic efficiency, antioxidant enzyme activity, and antioxidant substance content (e.g., lycopene, ascorbic acid) in tomatoes, thereby improving fruit quality and yield. Under salt stress, Zhang et al. (2023) demonstrated that PHs induce metabolic reprogramming in tomatoes, primarily affecting secondary metabolite, lipid, fatty acid, and phytohormone biosynthesis pathways. PHs also promote plant growth via nitrogen metabolism and carbon-nitrogen balance pathways, stimulating nitrogen uptake and utilization efficiency to enhance growth rate and biomass (Colla et al., 2017b; Cristofano et al., 2021; Choi et al., 2022). Exogenous PH application elevated soluble sugar content in tomato and wheat seedlings (Ertani et al., 2017; Genc and Atici, 2019) and upregulated the expression of amino acid transporter genes (e.g., AAT1 for glutamate, aspartate, and isoleucine) in tomato roots and leaves (Sestili et al., 2018). Additionally, PHs modulate phytohormonal pathways (e.g., gibberellin, auxin) to enhance seedling length, leaf area, dry matter accumulation, and photosynthetic efficiency, ultimately boosting crop productivity (Polo and Mata, 2018; Ceccarelli et al., 2021; Kisvarga et al., 2022). Carillo et al. (2022) showed that legume-derived PHs at 2.5-5.0 mL/L improved nutrient utilization, root dry weight, and leaf area in lettuce, with similar yield-enhancing effects observed in rice, maize, rapeseed, and wheat, manifested as increased plant height, root elongation, biomass, and chlorophyll content (Capo et al., 2023; Ujvári et al., 2023; Ancín et al., 2024; Mutlu-Durak et al., 2024). Furthermore, PHs mitigate biotic and abiotic stresses (e.g., high temperature, drought, salinity) during critical growth stages by regulating osmoregulation processes and activating antioxidant enzymatic systems (Cardarelli et al., 2022; Ma et al., 2022; Shahzad et al., 2023). For example, plant-derived PHs reduced yield losses in soybean under combined heat and drought stress (Mamatha et al., 2023) and enhanced drought tolerance in plants by scavenging reactive oxygen species and maintaining cellular redox balance (Francesca et al., 2020; Zuzunaga-Rosas et al., 2024; Kaya, 2025).

The global temperature has exhibited a persistent upward trend, with extreme high temperatures exerting detrimental effects on rice growth, yield, and quality (Krishnan et al., 2011). Projections indicate that a 1 °C increase in temperature during the rice growing season could result in a 10% reduction in yield (van Oort and Zwart, 2018). Rice demonstrates sensitivity to elevated temperatures (> 35 °C) across all developmental stages, including germination, vegetative growth, and reproductive phases (Li et al., 2023). Specifically, during the reproductive growth period, high temperatures lead to significant declines in seed setting rates and ultimately reduce grain yield (Jiang et al., 2020). High-temperature stress profoundly disrupts the physiological homeostasis of rice, particularly by inhibiting the enzymatic activity of key reactive oxygen species (ROS)-scavenging enzymes, such as superoxide dismutase (SOD), peroxidase (POD), and catalase (CAT) (Zhao et al., 2018a). Notably, enhancing the activities of CAT and SOD has been shown to improve heat tolerance in rice (Qiao et al., 2015). Under severe heat stress, the accumulation of malondialdehyde (MDA) increases, thereby impairing the structural and functional integrity of proteins and nucleic acids (Chakraborty and Bhattacharjee, 2015). As such, electrolyte leakage, transcript abundance of antioxidant genes, antioxidant enzyme activities, and MDA content serve as widely recognized indicators of membrane lipid peroxidation and oxidative damage, simultaneously reflecting the thermotolerance of plants (Higashi and Saito, 2019). Furthermore, high-temperature stress has been shown to disrupt photosynthetic efficiency, carbohydrate metabolism, and phytohormonal balance in plants (Huang et al., 2021; Xu et al., 2021). Through prolonged natural and artificial domestication, rice has evolved sophisticated adaptive mechanisms to mitigate heat stress damage and maintain normal growth. Current evidence highlights the involvement of multiple signaling pathways in the perception and transduction of heat stress signals in rice, including calcium ions (Ca^2+^), ROS, hydrogen peroxide (H_2_O_2_), protein kinases, and plant hormones (Kan and Lin, 2021; Saini et al., 2022). Concurrently, a diverse array of heat shock proteins (HSPs) and transcription factors, such as members of the MYB, NAC, WRKY, and AP2/ERF families, play critical roles in mediating transcriptional regulatory responses to high-temperature stress (Vaseva et al., 2022). Notably, pretreatment with protein hydrolysates (PHs) has been demonstrated to induce a cascade of stress-responsive mechanisms in plants, including enhanced antioxidant enzyme activities, accumulation of polyphenolic compounds, and increased biosynthesis of osmoregulatory substances (e.g., proline), thereby augmenting their overall stress resilience (Shukla et al., 2019; Vaseva et al., 2022).

Although proteolytic products (PHs) have shown substantial potential in agriculture, their complex biological characteristics and variable efficacy across systems remain poorly understood, with unclear mechanisms of action and suboptimal utilization strategies. PH efficacy is influenced by multiple interacting factors, including biological origin, target crop species, application dosage, timing, and biochemical composition (Colla et al., 2015; Carillo et al., 2025). While existing literature documents PH-mediated growth promotion in rice, the scientific basis for these effects remains incompletely elucidated due to the chemical complexity of PH formulations. Currently, research on soybean PH has primarily focused on dicotyledonous plants (e.g., tomato). These compounds have demonstrated application potential in promoting crop growth, increasing yield, and enhancing stress tolerance. However, there remains a significant gap in research regarding their effects on monocotyledonous plants. Therefore, investigating whether soybean PH exhibit similar application potential in rice and elucidating their underlying mechanism of action will provide a scientific basis for improving yield and ensuring stable production in rice cultivation. This study aims to by systematically evaluating a commercial soybean-derived PH (“Lifenggu”) (composed of small peptides, free amino acids, carbohydrates, and mineral elements) and focuses on the following core parameters to fill this gap: growth phenotypic parameters, the dose-effect relationships of soybean-derived PH regulators on rice phenotypic traits at key growth stages (seedling and booting stage), including leaf chlorophyll content (SPAD value), seedling length, seedling weight, yield, and yield-related traits. Physiological function parameters, effects of soybean PH on nitrogen metabolism enzyme activities (e.g., nitrate reductase (NR), Glutamine Synthetase (GS)), antioxidant enzyme activities (e.g., SOD, POD), proline content, and MDA content in rice. Molecular regulation parameters, under high-temperature stress, the transcriptomic expression characteristics (differentially expressed genes and pathways) involved in the regulation of rice growth by soybean-derived pH regulators. Field application parameters, The effects of soybean-derived pH regulators on leaf SPAD values, yield, and yield-related traits across multiple locations (15 experimental sites) and rice varieties. This provides valuable information for the application of plant growth regulators in rice production systems.

## 2 Materials and methods

### 2.1 Plant material, growth conditions, and treatments

The indoor experiment utilized the indica-japonica hybrid rice cultivar “Jia You Zhe Ke 3” as the test material. In Experiment 1 (seedling stage) varying concentrations of protein hydrolysate (PH, trade name “Lifenggu”) were applied and in Experiment 2 (herbicide stress), the optimal “Lifenggu” concentration (determined from Experiment 1) was applied. Seeds were sown at 46 seeds per 96-well cultivation box (1 L capacity), pre-cultivated in a growth chamber for 14 days, and experimental treatments were initiated after discarding underdeveloped seedlings. The growth chamber was set to a 14-h photoperiod/10-h dark cycle, with temperatures maintained at 28 °C during light periods and 26 °C during dark periods.

In Experiment 1, thirteen experimental groups (A-M) were established, with Group A as the untreated control (**Supplementary Table S1**). Following 21 days of treatment with different concentrations of the PH “Lifenggu”, phenotypic observations were recorded. Experiment 2 consisted of four treatment groups: control (CK), herbicide-only (H), optimal “Lifenggu” concentration (L), and “Lifenggu” combined with herbicide (L+H) (**Supplementary Table S2**). Phenotypic assessments were conducted after 7 days of treatment. Specifically, the L+H group received “Lifenggu” for 2 days prior to herbicide application for 7 days. Notably, the L and H groups were initiated simultaneously with the herbicide application in the L+H group to ensure temporal consistency across comparative treatments. The herbicide used was oxazolyl-chloropyrifos-cyhalofop at a concentration of 0.25 mL/L.

For Experiment 3 (spraying different concentrations of “Lifenggu” at the booting stage) (**Supplementary Table S3**) and Experiment 4 (high-temperature stress at 42 °C), indoor pot culture experiments were conducted. Rice seedlings were first cultivated in the field and then transplanted into plastic buckets. Each bucket contained three seedlings, which were treated at the booting stage. Ten replicate pots were assigned to each experimental group to ensure statistical reliability. Experiment 3 included six treatment groups (CK as the control, and C1-C5), with varying concentrations of “Lifenggu” applied via foliar spraying at the booting stage. In Experiment 4, the optimal “Lifenggu” concentration (determined from Experiment 3) was applied similarly. Twenty-four hours after treatment, potted plants were transferred to a high-temperature chamber set at 42 ± 0.5 °C. A control group remained under normal conditions throughout. Following 96 hours of high-temperature exposure, all plants were returned to normal environmental conditions for recovery.

Field trials were conducted across 15 experimental sites in various counties (cities/districts) of Jiangxi Province, including 1 early-season rice group, 9 mid-season rice groups, and 5 late-season rice groups (**Supplementary Table S4**). Each site included two treatments: non-application of “Lifenggu” (CK) and “Lifenggu” application (L). For the L treatment, “Lifenggu” was applied at 4500 mL·hm^-2^ by spraying the seedling bed 2-3 days before rice transplantation. Field spraying was performed in two stages: the first application at 1500 mL·hm^-2^ during the mid-to-late tillering stage (sealing period), and the second application at 1500 mL·hm^-2^ 7-10 days prior to jointing. All other agricultural practices followed local conventional management protocols. The CK treatment followed identical conventional management without “Lifenggu” application.

### 2.2 SPAD value measurement

Leaf chlorophyll content was quantified using a SPAD-502 chlorophyll meter at the widest section of the uppermost leaves of rice plants. In the seedling-stage experiment, measurements were taken from 30 plants per treatment; at the heading stage, 12 plants per treatment were measured; and in field experiments, 30 plants per treatment were assessed. Average values from these measurements were recorded as the SPAD values for each treatment. During measurement, leaves were positioned in the chlorophyll meter’s chamber, avoiding the main vein, and the leaf holder was secured to ensure horizontal placement and consistent measurement areas. Care was taken to minimize background reflection, leaf surface curvature, spectral fluctuations, and intra-leaf variations that could affect readings.

### 2.3 Determination of N-metabolism-related enzyme activities

Rice cultivar “Jia You Zhe Ke 3” was used as the experimental material. Seeds were soaked and germinated until radicles reached 2-3 mm in length, then sown in 96-well hydroponic boxes containing distilled water. After 14 days of cultivation under 28 °C/26 °C with a 14-h light/10-h dark cycle, treatments were applied. Untreated seedlings served as the control, while the experimental group was treated with 1 mL/L “Lifenggu” for 12h. Leaves and roots from both groups were harvested as detection samples, rapidly frozen in liquid nitrogen, and stored at −80 °C.

For the booting-stage experiment, seedlings were first cultivated in the field and then transplanted into plastic buckets with a volume of 20 liters (three seedlings per bucket). The transplantation was performed when the seedlings were 25 days old. Treatments were applied at the booting stage, with untreated plants as controls and treated plants sprayed with different concentrations of “Lifenggu” for 24h. Functional leaves from all groups were collected as test samples, processed with liquid nitrogen freezing, and stored at −80 °C.

Enzyme activities of nitrate reductase (NR), glutaminase (GLS), nitrite reductase (NiR), glutamate synthase (GOGAT), glutamate dehydrogenase (GDH), glutamine synthetase (GS), and ferredoxin-dependent glutamate synthase (Fd-GOGAT) were determined using commercial test kits according to the manufacturer’s instructions. The reagent kit was purchased from Beijing Solab Technology Co., Ltd. (NR reagent kit (BC0080), NiR reagent kit (BC1540), (ADS059TE0), GS reagent kit (BC0915), GLS reagent kit (BC1455), GDH reagent kit (BC1465), and GOGAT reagent kit (BC0075)). Fd-GOGAT reagent kit (JL-T0650) was purchased from Shanghai Qiyi Industrial Co., Ltd.

### 2.4 Determination of adversity-related physiological and biochemical indicators

The rice cultivar “Jia You Zhe Ke 3” was used as the experimental material. Seeds were soaked and germinated until radicles reached 2-3 mm in length, then sown in 96-well hydroponic boxes containing distilled water. After 14 days of cultivation under a 28 °C/26 °C temperature regime with a 14-h light/10-h dark cycle, treatments were applied. For herbicide stress experiments, samples were divided into two groups: herbicide-only treatment for 12h (H) and combined “Lifengu” + herbicide treatment for 12h (L+H). Leaves and roots from both groups were harvested as detection samples, rapidly frozen in liquid nitrogen, and stored at −80 °C.

For the booting-stage experiment, seedlings were first cultivated in the field and then transplanted into plastic buckets with a volume of 20 liters (three seedlings per bucket). The transplantation was performed when the seedlings were 25 days old. Treatments were applied at the booting stage as follows:

Treatment 1: Untreated plants served as controls, while plants sprayed with 2.5 mL/L “Lifengu” for 24h comprised the treatment group. Functional leaves were collected from both groups and stored as described above.
Treatment 2: After pretreatment with 2.5 mL/L “Lifengu” for 24h, potted plants were exposed to a high-temperature environment (42 ± 0.5 °C) for 12h. Control plants underwent heat stress without “Lifengu” pretreatment, while treated plants received both pretreatment and heat stress. Functional leaves from both groups were collected and stored at −80 °C.

The aforementioned samples were used to measure hydrogen peroxide (H_2_O_2_), malondialdehyde (MDA), and proline (Pro) content, as well as antioxidant enzyme activities [superoxide dismutase (SOD), peroxidase (POD), catalase (CAT)] using commercial test kits according to the manufacturers’ protocols. The SOD reagent kit (ADS-F-KY001), CAT reagent kit (ADS-W-KY002), POD reagent kit (ADS-F-KY003) and proline reagent kit (ADS-W-AJS004) were purchased from Jiangsu Adisun Biotechnology Co., Ltd. The MDA reagent kit (BC0020) and hydrogen peroxide reagent kit (BC3590) were purchased from Beijing Solabio Technology Co., Ltd.

### 2.5 Transcriptomic analysis

To investigate the biological regulatory networks underlying “Lifenggu”-mediated growth promotion and high-temperature tolerance in rice, transcriptomic profiling was performed using the rice cultivar “Jia You Zhe Ke 3”. Seedlings were first cultivated in the field and transplanted into plastic buckets (three seedlings per bucket), with treatments applied at the booting stage. Under normal environmental conditions (28 °C/26 °C, 14-h light/10-h dark cycle), untreated plants served as controls, while the experimental group comprised plants treated with 2.5 mL/L “Lifengu” solution for 24h. For heat stress conditions, samples pretreated with 2.5 mL/L “Lifengu” for 24h were exposed to a high-temperature environment (42 °C) for 12h. Untreated plants under heat stress served as controls, and pretreated plants subjected to heat stress were designated as the treatment group. Samples (functional leaves) were harvested, rapidly frozen in liquid nitrogen, and stored at −80 °C. Twelve RNA samples were prepared, including two experimental groups (normal and heat stress) with three biological replicates each (treated and control).

Total RNA (1 µg per sample) was used for library preparation using the NEBNext^®^ Ultra™ RNA Library Prep Kit for Illumina^®^ (NEB, USA) following the manufacturer’s protocol. Key steps included: Polyadenylated mRNA enrichment via poly-dT magnetic beads; RNA fragmentation using divalent cations in 5× NEBNext First Strand Synthesis Buffer; First-strand cDNA synthesis with random hexamers and M-MuLV Reverse Transcriptase (RNase H^-^); Second-strand cDNA synthesis using DNA Polymerase I and RNase H; End repair, 3′ adenylation, and ligation of NEBNext hairpin adaptors with unique dual-indexing; Size selection (250–300 bp) via AMPure XP bead purification; USER Enzyme treatment (37 °C, 15min; 95 °C, 5min) and PCR amplification with Phusion High-Fidelity DNA Polymerase; Final library quality verified on an Agilent Bioanalyzer 2100. Raw sequencing data were processed using fastp (v0.19.3) to trim adapters and filter low-quality reads (discarded if N-content > 10% or > 50% bases with Q ≤ 20). Clean reads were aligned to the reference rice transcriptome (MSU Rice Genome Annotation Project, accessed 8 June 2024). Transcript abundance was quantified as FPKM (Fragments Per Kilobase of transcript per Million mapped reads) using featureCounts (v1.6.2). Differential expression analysis was performed with DESeq2 (v1.22.1), identifying significantly differentially expressed genes (DEGs) using Benjamini-Hochberg FDR correction (*padj*<0.05 and |log__2__^fold-change^| ≥ 1). Functional enrichment of DEGs was conducted via hypergeometric tests for Gene Ontology (GO) terms and Kyoto Encyclopedia of Genes and Genomes (KEGG) pathways. RNA sequencing and initial bioinformatics were conducted by Wuhan Maiwei Metabolism Biotechnology Co., Ltd. (Wuhan, China).

### 2.6 Trait measurements

Yield-related traits were measured at plant maturity. Tiller number, panicle length, total grains per panicle, filled grains per panicle, 1000-grain weight, seed setting rate, and grain yield per plant were recorded. For 1000-grain weight, 100 plump seeds were randomly selected and weighed, with three replicates, and the average value was converted to 1000-grain weight. Grain yield per plant was defined as the total weight of filled grains per plant. For plot-level rice yield determination, all harvested grains within designated 10 × 10m areas were weighed. Three such areas were randomly selected, and the average yield was calculated and converted to yield per hectare.

### 2.7 Data analysis

The phenotypic data obtained in this study were statistically processed using Microsoft Excel. Subsequent data analysis was performed using IBM SPSS Statistics 26.0 (SPSS Inc., Chicago, IL, USA). Duncan’s multiple range test was employed to assess significant differences (*P*<0.01) in phenotypic values among treatments in the indoor experiments. Independent samples t-tests (*P* <0.05) were used to detect differences in physiological indices between treatment groups and differences in phenotypic data from field experiments.

## 3 Result

### 3.1 Field application of “Lifenggu” in rice production

**Figure 1:**
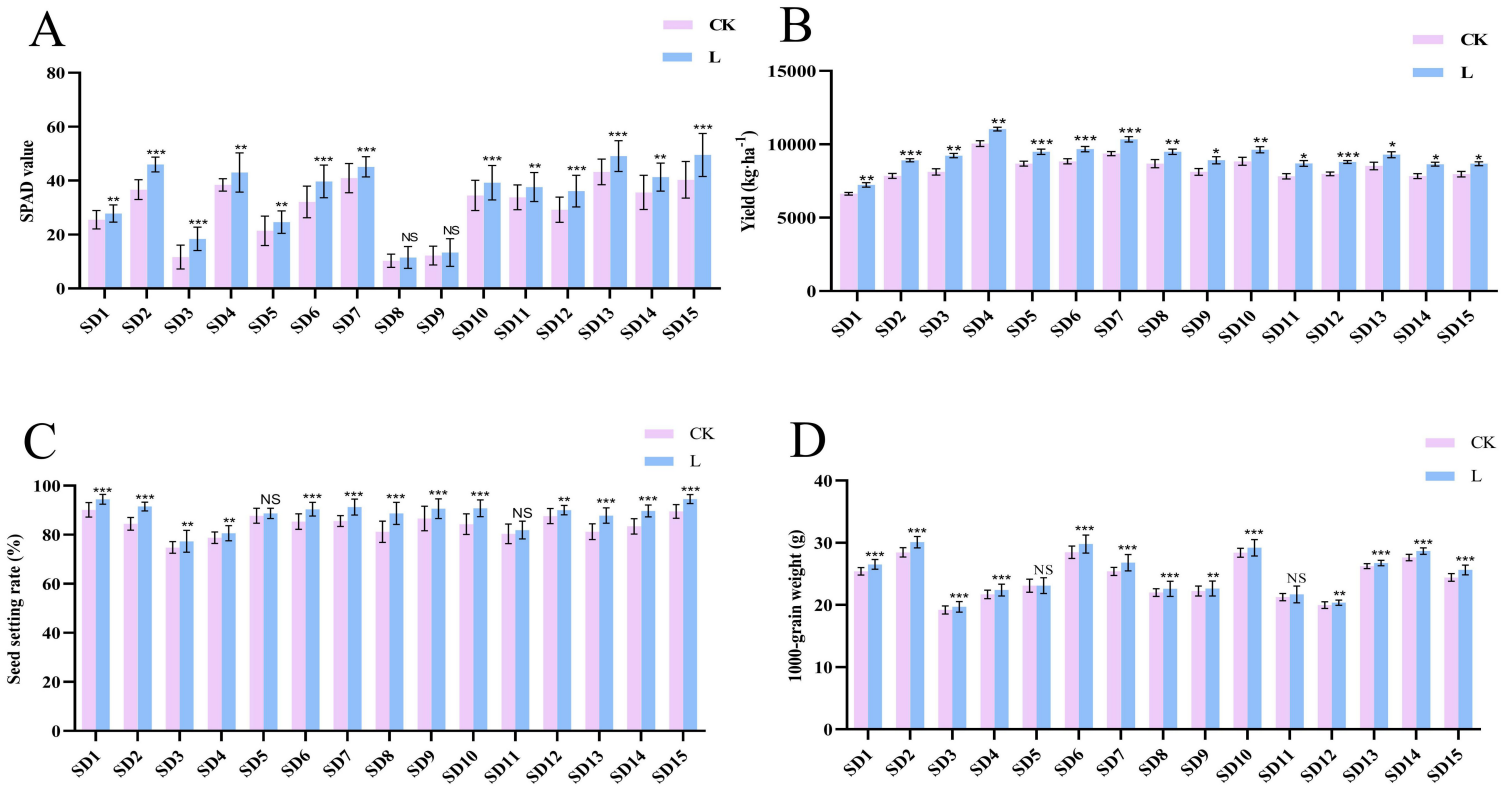
Analysis of SPAD values, yield, and yield components across 15 experimental sites following “Lifenggu” treatment. **(A)** SPAD values in the “Lifenggu”-treated group (L) versus the control group (CK) across 15 sites. **(B-D)** Yield and yield components (total grains per panicle, filled grains per panicle, seed setting rate, 1000-grain weight, yield per mu) in L versus CK across 15 sites. In all panels, on the x-axis, SD1 to SD15 represent 15 experimental sites, where SD1 denotes the early-season rice group, SD2 to SD10 represent the mid-season rice groups, and SD11 to SD15 correspond to the late-season rice groups. Detailed information for SD1 to SD15 is provided in **Supplementary Table S4**; *, **, and *** denote significant differences at *P*<0.05, *P*<0.01, and *P*<0.001, respectively; NS indicates no significant difference.

To investigate the effects of “Lifenggu” on rice productivity, carried out field trials. The trials included one early-season rice group, nine mid-season rice groups, and five late-season rice groups. Results showed that in the early-season rice group (SD1), all yield-related traits except panicle length significantly increased following “Lifenggu” application. Specifically, total grains per panicle, filled grains per panicle, seed setting rate, 1000-grain weight, yield per plant, yield per hectare, and SPAD value increased by 7.9%, 13%, 4.8%, 4.4%, 13%, 9.1%, and 9.1%, respectively (**Figure 1**; **Supplementary Table S5**). In mid-season rice groups (SD2-SD10), “Lifenggu” spraying significantly increased tiller number at three sites (SD2, SD5, and SD7). SPAD values showed no significant changes at sites SD8 and SD9 but increased significantly at the remaining seven sites, with an average increase of 20%. Notably, site SD3 exhibited the most dramatic enhancement (58%). Across all sites, five key yield parameters (including total grains per panicle, filled grains per panicle, seed setting rate, yield per plant, and grain yield per hectare) showed significant average increases of 8.1%, 14%, 5.5%, 12%, and 11%, respectively. Site SD3 had the largest increase in total grains per panicle (17.65%), Site SD8 had the largest increase in seed setting rate (9.2%), while SD2 showed the highest improvements in other metrics: 21% (filled grains per panicle), 26% (yield per plant), and 14% (grain yield per hectare). 1000-grain weight increased significantly at eight sites (excluding SD5), with an average gain of 3.2%, and site SD2 showed the maximum increase (5.8%) (**Figure 1**; **Supplementary Table S5**). In late-season rice groups (SD11-SD15), “Lifenggu” application significantly increased SPAD values and grain yield per hectare across all sites, with average gains of 17% and 9.9%, respectively. Site SD12 exhibited the largest SPAD increase (24%), while SD11 showed the highest yield enhancement (11%). Tiller number increased significantly at SD11 and SD12, with moderate but non-significant increases at SD13-SD15. Panicle length increased significantly only at SD11, with no significant changes at SD12-SD15. Total grains per panicle rose significantly at SD13-SD15 (mean +5.1%). Filled grains per panicle, seed setting rate, and 1000-grain weight showed significant improvements across SD12-SD15, with mean increases of 11%, 5.2%, and 3.0%, respectively. Notably, SD13 had the largest increases in filled grains per panicle (16%) and seed setting rate (8.1%), while SD15 showed the highest 1000-grain weight improvement (5.0%) (**Figure 1**; **Supplementary Table S5**).

In summary, “Lifenggu” application significantly enhanced SPAD values and yield-related agronomic traits in rice across all growth seasons, with most improvements reaching statistical significance. These results suggests that the application of “Lifengu” promoted rice growth, yield improvement and SPAD values. Among early-, mid-, and late-season rice groups, mid-season rice demonstrated the most pronounced responses. Across all 15 experimental sites, site SD2 exhibited the highest overall efficacy of “Lifenggu”.

### 3.2 Effects of different “Lifenggu” concentrations on rice seedlings

**Figure 2:**
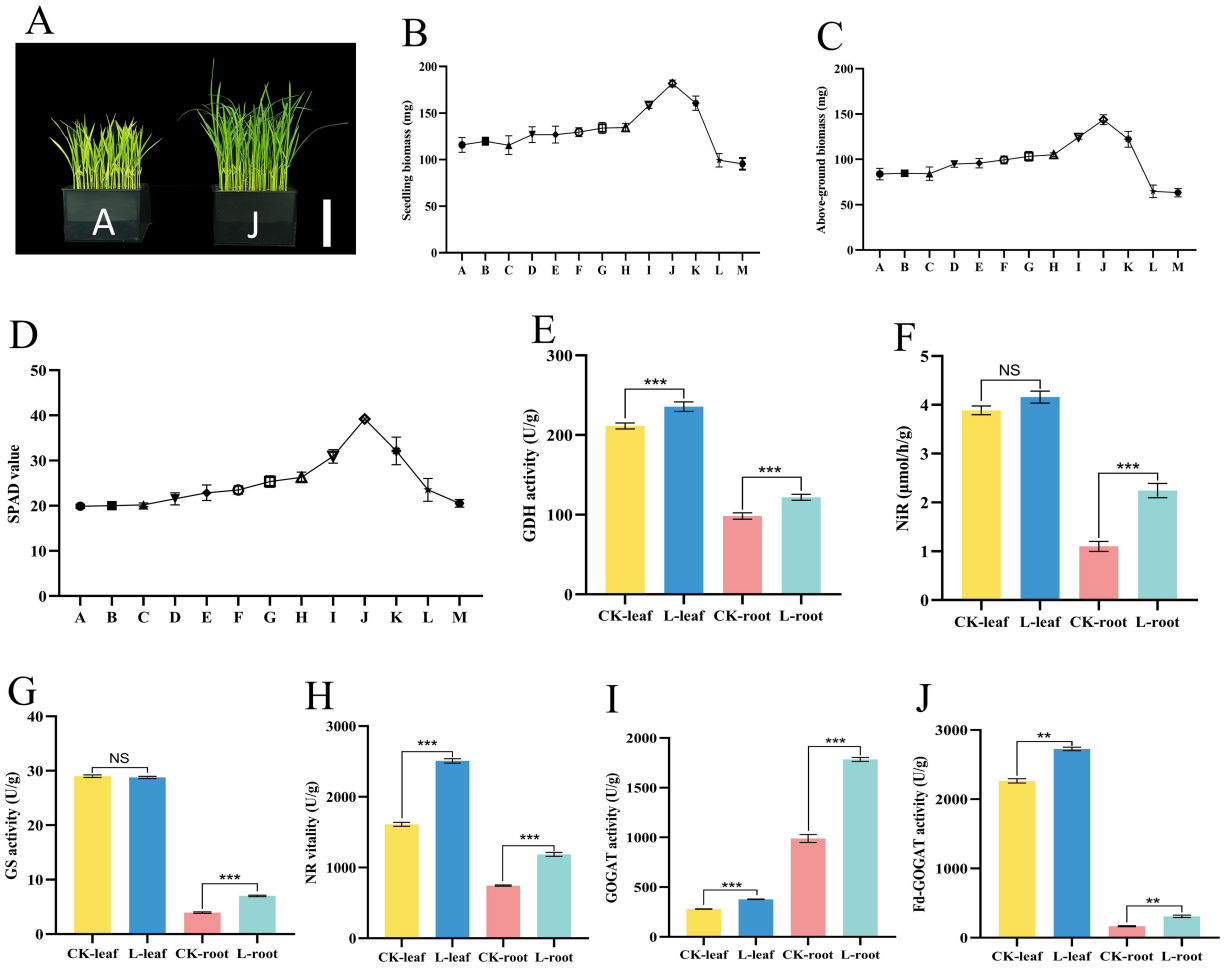
Analysis of phenotypic traits and physiological indicators in rice seedlings treated with different “Lifenggu” concentrations. **(A)** Phenotypic comparison between the optimal concentration treatment group (J) and control group (A), bar = 10cm. **(B**-**D)** Seedling biomass, above-ground biomass, and SPAD values across “Lifenggu” concentrations. In panels **(B‑D)**, Groups A through M represent the experimental groups treated with different concentrations of “Lifenggu”, where Group A serves as the control group. The concentrations of “Lifenggu” added are as follows: A (0 mL/L), B (0.06 mL/L), C (0.08 mL/L), D (0.13 mL/L), E (0.17 mL/L), F (0.25 mL/L), G (0.33 mL/L), H (0.50 mL/L), I (0.67 mL/L), J (1 mL/L), K (1.33 mL/L), L (2 mL/L), and M (3.33 mL/L). **(E-J)** Nitrogen metabolism-related enzyme activities [glutamate dehydrogenase (GDH), nitrite reductase (NiR), glutamine synthetase (GS), nitrate reductase (NR), glutamate synthase (GOGAT), and ferredoxin-dependent glutamate synthase (Fd-GOGAT)] in above-ground tissues and roots of control (A) and optimally treated (J) seedlings. *CK-leaf* and *CK-root* denote above-ground tissues and roots of the control group (Group A), respectively; *L-leaf* and *L-root* represent above-ground tissues and roots after 12-hour treatment with the optimal concentration (Group J), respectively. In panels **(E‑J)**, ** and *** denote significant differences at *P*<0.01 and *P*<0.001, respectively; NS indicates no significant difference.

To determine the optimal “Lifenggu” concentration for rice seedling growth, this study applied varying concentrations of the protein hydrolysate to seedlings via adding supplementation. Results showed that seedling biomass in treatment groups B-J increased in a concentration-dependent manner. However, further increases in concentration (groups K-M) led to a decline in biomass accumulation, with significant reductions of 14% and 17% observed in groups L and M, respectively, compared to the control group (A). The maximum growth promotion (57% biomass increase relative to the control) was achieved at 1 mL/L (Group J) (**Figure 2B**; **Supplementary Table S6**). Seedling length and above-ground biomass exhibited dose-response patterns consistent with overall seedling biomass dynamics across “Lifenggu” concentrations. The maximum growth promotion was observed in Treatment J (1 mL/L), with seedling length and above-ground biomass increasing by 20% and 72%, respectively, compared to Control A. In contrast, Treatments L (2 mL/L) and M (3.33 mL/L) showed significant reductions relative to the control: Group L had 3.9% and 23% decreases in seedling length and above-ground biomass, while Group M exhibited 5.5% and 24% reductions, respectively (**Figures 2B, C**; **Supplementary Table S6**). Root length and root biomass showed no consistent trends with increasing “Lifenggu” concentration (**Supplementary Figures S1C, D**). Nevertheless, Treatment J (1 mL/L) still induced the greatest growth promotion, with root length and biomass increasing by 11% and 12%, respectively (**Supplementary Table S6**). SPAD values across Groups A-M followed a pattern consistent with seedling biomass: increasing initially and then decreasing, with a peak at Treatment J (1 mL/L) that showed a 97% increase relative to Group A. No significant differences in SPAD values were observed between high-concentration Groups L/M and the control (Group A) (**Figure 2D**; **Supplementary Table S6**).

Rice seedlings treated with varying “Lifenggu” concentrations exhibited auxin-like growth responses: high concentrations (L and M) inhibited seedling growth, while low-to-moderate concentrations (G-K) promoted growth. Very low concentrations (B-F), however, did not induce statistically significant effects. These results indicate that low-dose “Lifenggu” supplementation can enhance rice seedling growth. Among all treatments, 1 mL/L “Lifenggu” (Group J) elicited the most pronounced growth promotion across all metrics, achieving peak values in biomass, seedling length, and SPAD values relative to the control (Group A), thus representing the optimal concentration for the seedling stage (**Figure 2A**; **Supplementary Figure S1A**). In contrast, high-concentration treatments in Groups L (2 mL/L) and M (3.33 mL/L) significantly inhibited seedling growth, underscoring the critical role of protein hydrolysate (PH) dosage in determining growth outcomes.

To investigate the physiological mechanisms by which “Lifenggu” promotes rice seedling growth, above-ground tissues and roots were sampled from the optimal concentration group (J) and control group (A) for measuring seven nitrogen (N) metabolism-related indicators. In above-ground parts, activities of nitrate reductase (NR), glutamate synthase (GOGAT), and ferredoxin-dependent glutamate synthase (Fd-GOGAT) in Group J were significantly higher than those in Group A (*P*<0.01), with increases of 56%, 35%, and 20%, respectively (**Figures 2H–J**; **Supplementary Table S7**). Conversely, activities of glutamate dehydrogenase (GDH), glutaminase (GLS), glutamine synthetase (GS), and nitrite reductase (NiR) showed no significant changes in Group J (**Figures 2E–G**; **Supplementary Figure S2E**). In roots, GLS was the only parameter without significant differences between Groups A and J (**Supplementary Figure S2E**). All other measured indices in Group J exhibited significant increases, with most exceeding 50% compared to the control. Notably, NiR activity showed the most pronounced increase (105%) (**Supplementary Table S7**). The changes in N metabolic enzyme activities were consistent with improvements in seedling biomass, length, and above-ground weight, collectively contributing to enhanced overall biomass. These results suggest that “Lifenggu” application at the seedling stage may enhance nitrogen uptake and utilization efficiency by increasing the activities of nitrogen metabolic enzymes, potentially contributing to the promotion of rice seedling growth. Further studies are needed to directly verify the effects on nitrogen uptake and utilization efficiency.

### 3.3 “Lifenggu” enhances herbicide resistance in rice seedlings

**Figure 3:**
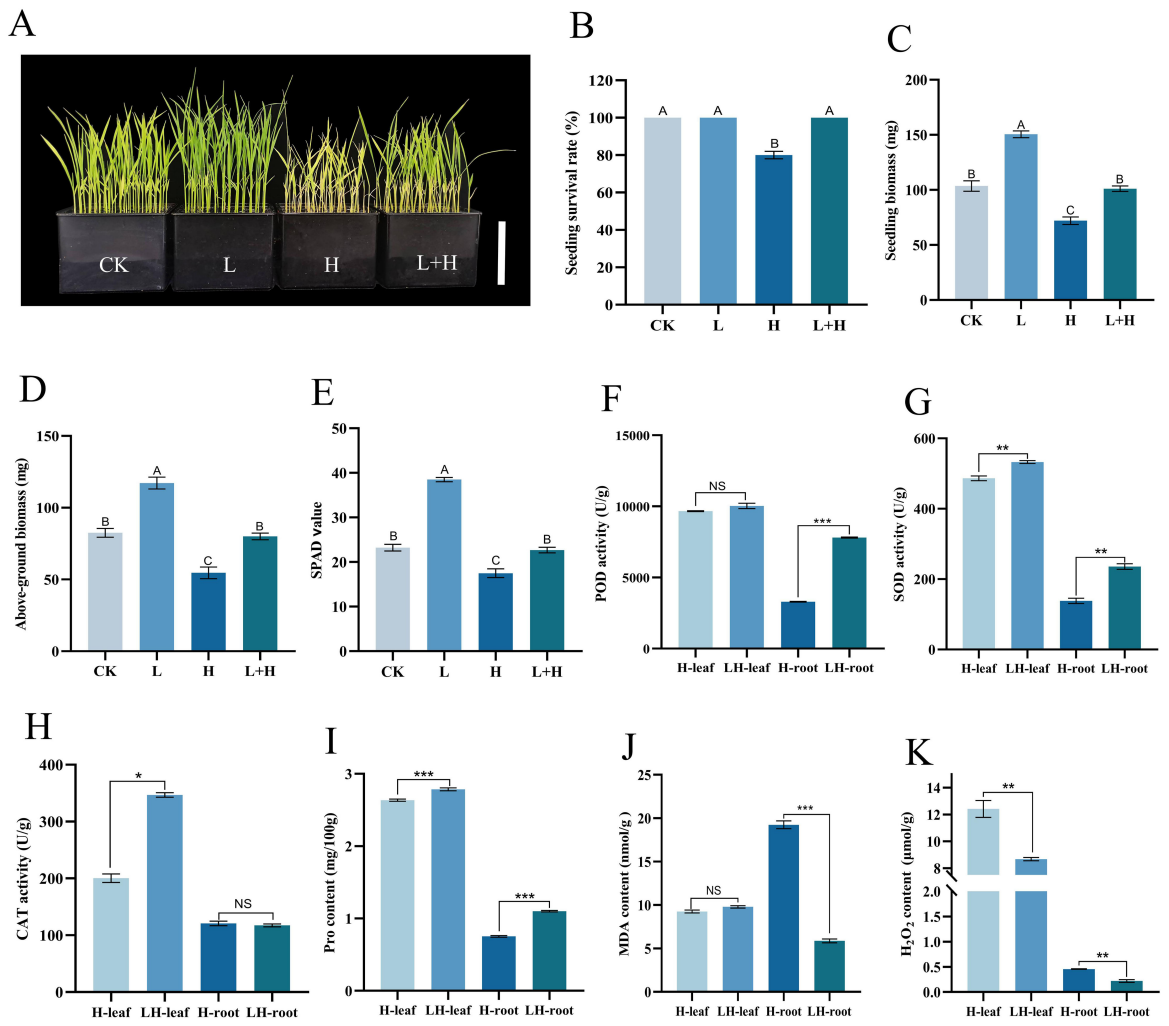
Analysis of phenotypic traits and physiological indicators in rice seedlings under herbicide stress with optimal “Lifenggu” concentration. **(A)** Phenotypic comparison among control (CK), “Lifenggu”-only (L), herbicide-only (H), and “Lifenggu”+herbicide (L+H) treatment groups, bar = 10cm. **(B-E)** Phenotypic traits across treatments. In panels **(B-E)**, the concentrations of “Lifenggu” and herbicide added are as follows in four experimental groups: CK (0 mL/L+ 0 mL/L), L (1 mL/L+ 0 mL/L), H (0 mL/L+ 0.25 mL/L), L+H (1 mL/L+ 0.25 mL/L). **(B)** Seedling survival rate, **(C)** Seedling biomass, **(D)** Above-ground biomass, **(E)** SPAD values. In panels **(B-E)**, the concentrations of "Lifenggu" and herbicide added are as follows in four experimental groups: CK (0 mL/L+ 0 mL/L), L (1 mL/L+ 0 mL/L), H (0 mL/L+ 0.25 mL/L), L+H (1 mL/L+ 0.25 mL/L), and different capital letters indicate significant differences (*P*<0.01) among treatments, while shared letters denote no significant differences. **(F-K)** Stress-related parameters in above-ground tissues and roots of H (add herbicide-only at 0.25 mL/L) vs. L+H (add "Lifenggu" at 1 mL/L+herbicide at 0.25 mL/L) treatment groups: **(F)** Peroxidase (POD) activity, **(G)** Superoxide dismutase (SOD) activity, **(H)** Catalase (CAT) activity, **(I)** Proline (Pro) content, **(J)** Malondialdehyde (MDA) content, **(K)** Hydrogen peroxide (H_2_O_2_) content. In panels (**F–K)**, On the horizontal axis, H-leaf and H-root denote above-ground tissues and roots of the H treatment groups, respectively; LH-leaf and LH-root represent above-ground tissues and roots of the L+H treatment groups, respectively; *, **, and *** denote significant differences at *P*<0.05, *P*<0.01, and *P*<0.001, respectively; NS denotes non-significant.

To investigate the potential of “Lifenggu” in alleviating herbicide-induced damage to rice seedlings, this study evaluated agronomic and physiological parameters in seedlings co-treated with the optimal “Lifenggu” concentration (1 mL/L) and herbicide (0.25 mL/L). At the end of the experimental period (7 days after treatment, 7 DAT), growth parameters including SPAD value, seedling biomass, seedling length, root length, and above-ground/root biomass were measured (**Supplementary Table S8**). Seedlings treated with herbicide alone (H) had an 80% survival rate, whereas those co-treated with “Lifenggu” and herbicide (L+H) exhibited 100% survival (**Figures 3A, B**). Herbicide treatment alone (H) significantly reduced SPAD value, seedling length, seedling biomass, above-ground biomass, and root biomass (*P*<0.01) compared to other groups. In contrast, the L+H group showed no significant differences from the untreated control (CK) in these parameters and had significantly higher values than the H group. Notably, the “Lifenggu”-only treatment group (L) exhibited significantly higher phenotypic values than CK (*P*<0.01) (**Figures 3C–E**; **Supplementary Figures S2AC**). These results demonstrate that optimal “Lifenggu” concentration mitigates herbicide phytotoxicity and promotes seedling growth by enhancing stress resistance and maintaining biomass accumulation. Specifically, above-ground biomass showed a marked increase following “Lifenggu” treatment, highlighting its role in counteracting herbicide-induced growth inhibition.

To investigate how “Lifenggu” mitigates herbicide stress, aboveground tissues and roots from the herbicide-only (H) and “Lifenggu”+herbicide (L+H) groups were analyzed for six stress-related indicators: peroxidase (POD) activity, superoxide dismutase (SOD) activity, catalase (CAT) activity, malondialdehyde (MDA) content, hydrogen peroxide (H_2_O_2_) content, and proline (Pro) content (**Supplementary Table S9**). In above-ground tissues, L+H treatment significantly reduced H_2_O_2_ content by 30.03% compared to H treatment (**Figure 3K**). SOD activity, CAT activity, and Pro content were significantly higher in L+H than in H (*P*<0.01), increasing by 9.5%, 73%, and 5.7%, respectively (**Figures 3G–I**; **Supplementary Table S9**). POD activity showed no significant change (**Figure 3F**), while MDA content trended downward albeit non-significantly (**Figure 3J**). In roots, L+H treatment significantly decreased H_2_O_2_ and MDA content by 51% and 223% (*P*<0.01), respectively, compared to H treatment (**Figures 3K, J**; **Supplementary Table S9**). In root, POD activity, SOD activity, and Pro content in L+H were significantly elevated by 137%, 71%, and 47%, respectively (**Figures 3F, G, I**; **Supplementary Table S9**), while CAT activity showed no significant difference (**Figure 3H**). These results suggest that the improved herbicide resistance observed in “Lifenggu”-treated seedlings is associated with modulated antioxidant enzyme activities and osmolyte accumulation. This correlation may contribute to a reduction in ROS-induced damage and help maintain cellular homeostasis under herbicide stress.

### 3.4 Effects of different “Lifenggu” concentrations on rice at the booting stage

**Figure 4:**
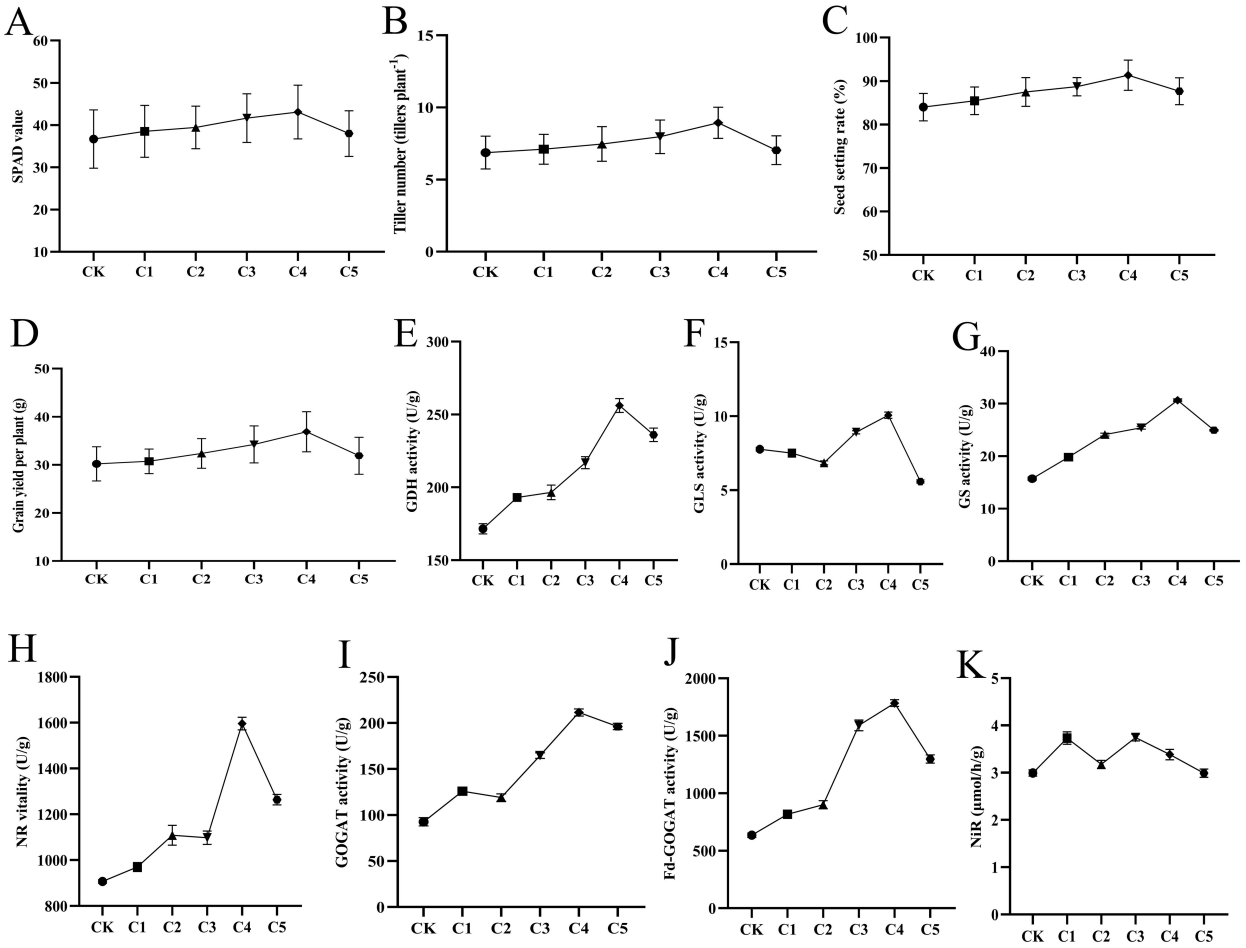
Analysis of phenotypic traits and physiological indicators under different “Lifenggu” concentrations at the booting stage. **(A-D)** Statistical analysis of SPAD values, tiller number, seed setting rate, and grain yield per plant in treatment groups C1-C5 versus the control (CK). **(E-K)** Activities of seven nitrogen (N) metabolism-related enzymes in treatment groups C1-C5 versus CK: **(E)** Glutamate dehydrogenase (GDH), **(F)** Glutaminase (GLS), **(G)** Glutamine synthetase (GS), **(H)** Nitrate reductase (NR), **(I)** Glutamate synthase (GOGAT), **(J)** Ferredoxin-dependent glutamate synthase (Fd-GOGAT), **(K)** Nitrite reductase (NiR). In all panels, Groups CK and C1-C5 represent the experimental groups treated with different concentrations of “Lifenggu”, where Group CK serves as the control group. The concentrations of “Lifenggu” sprayed are as follows: CK (0 mL/L), C1 (0.31 mL/L), C2 (0.67 mL/L), C3 (1.25 mL/L), C4 (2.50 mL/L), C5 (5 mL/L).

To determine the optimal “Lifenggu” concentration for rice during the booting stage, pot experiments with foliar spray applications were conducted across varying concentrations. Data analysis showed that in treatment groups C1-C5, SPAD values, tiller number, total grains per panicle, filled grains per panicle, seed setting rate, 1000-grain weight, and grain yield per plant initially increased with rising concentrations (C1-C4) before decreasing at C5 (**Figures 4A–D**; **Supplementary Figures S3CE**). The C4 treatment (2.5 mL/L) achieved peak values for all parameters, significantly outperforming the control (CK) and other treatments. Specifically, these parameters increased by 17% (SPAD), 29% (tiller number), 6.8% (total grains per panicle), 17% (filled grains per panicle), 8.7% (seed setting rate), 4.5% (1000-grain weight), and 22% (grain yield per plant) compared to CK (**Supplementary Table S10**). While C5 showed reduced efficacy relative to C4, it still exhibited significant improvements over CK. Panicle length remained unchanged across all treatments (**Supplementary Figure 3B**). These results suggests that the application of “Lifengu” promoted rice growth and yield improvement, which may be associated with the enhancement of SPAD values. The C4 treatment (2.5 mL/L) demonstrated the most pronounced growth improvements and highest yield enhancement relative to CK, establishing it as the optimal concentration for booting-stage application.

To elucidate the physiological basis of “Lifenggu” effects during rice booting, activities of seven nitrogen (N) metabolism enzymes were analyzed across six treatment groups. In C1-C5 groups, glutamate dehydrogenase (GDH), glutamine synthetase (GS), nitrate reductase (NR), glutamate synthase (GOGAT), and ferredoxin-dependent glutamate synthase (Fd-GOGAT) activities exhibited a dose-response pattern: increasing from C1-C4 before declining at C5, with peak values in C4 (**Figures 4E, G–J**). Although GOGAT activity in C2 was slightly lower than C1, it remained significantly higher than the control (CK) (**Figure 4I**; **Supplementary Table S11**). In contrast, glutaminase (GLS) and nitrite reductase (NiR) activities showed no clear concentration-dependent trends (**Figures 4F, K**). Specifically, GLS and NiR activities in C1, C3, and C4 were significantly higher than CK, while C2 and C5 showed reduced GLS activity (relative to CK) and no significant NiR changes (**Supplementary Table S11**). In the C4 treatment (2.5 mL/L), GDH, GLS, GS, NR, GOGAT, Fd-GOGAT, and NiR activities increased by 49%, 30%, 95%, 76%, 128%, 181%, and 13% compared to CK, respectively (**Supplementary Table S11**). Except for NiR, all enzymes reached maximal activity in C4, with significant enhancements over both CK and other treatment groups (*P*<0.01). The correlation between N enzyme activities and yield-related traits was evident: C4 exhibited the strongest synergistic increases in both parameters. These results suggests that the application of “Lifengu” promoted rice growth and yield improvement, which may be associated with the enhancement of SPAD values and nitrogen metabolism enzyme activities. The C4 treatment (2.5 mL/L) was confirmed as the optimal concentration for booting-stage application (**Supplementary Figure S2A**).

### 3.5 “Lifenggu” enhances heat tolerance in rice

**Figure 5:**
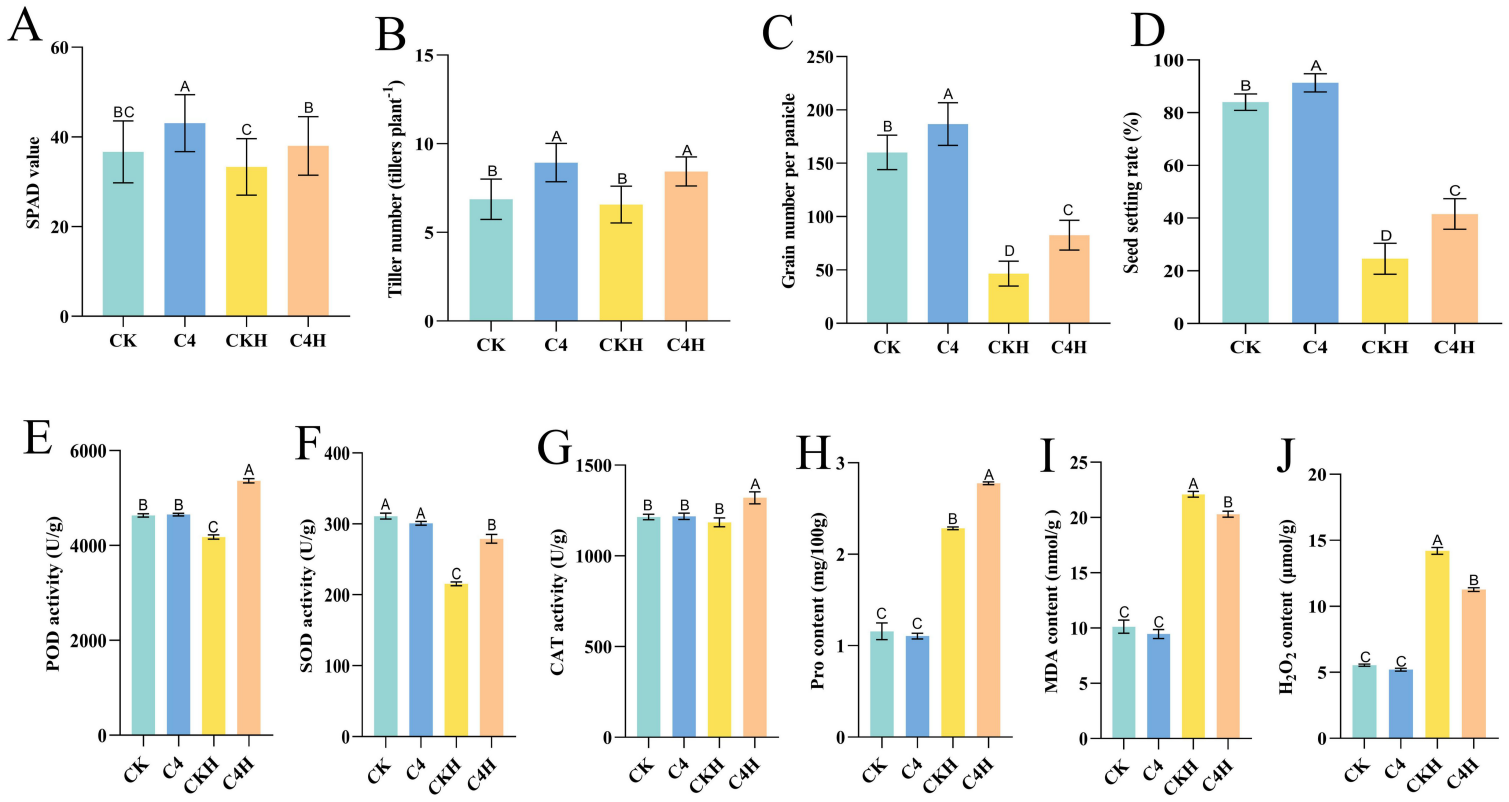
Analysis of “Lifenggu” foliar application on high-temperature stress responses and physiological parameters at the booting stage. **(A-D)** Phenotypic traits under normal and heat stress conditions with/without “Lifenggu” treatment: **(A)** SPAD values, **(B)** Tiller number, **(C)** Total grains per panicle, **(D)** Seed-setting rate. In panels **(A-D)**, different capital letters indicate significant differences (*P*<0.01) among treatments, while shared letters denote no significant differences. **(E-J)** Physiological indicators under normal conditions and high-temperature stress: **(E)** Peroxidase (POD) activity, **(F)** Superoxide dismutase (SOD) activity, **(G)** Catalase (CAT) activity, **(H)** Proline (Pro) content, **(I)** Malondialdehyde (MDA) content, **(J)** Hydrogen peroxide (H_2_O_2_) content. In all panels on the x-axis, CK denotes the blank control group under normal conditions, C4 represents the test group under normal conditions sprayed with Lifenggu (2.5 mL/L), CKH indicates the blank control group under high-temperature conditions (42°C), and C4H corresponds to the test group under high-temperature conditions (42°C) sprayed with Lifenggu (2.5 mL/L). Different capital letters indicate significant differences (P < 0.01) among treatments, while shared letters denote no significant differences.

To investigate whether “Lifenggu” improves thermotolerance in rice, plants at the booting stage were sprayed with the optimal concentration (2.5 mL/L) before high-temperature treatment (42 °C). Under normal conditions, all measured phenotypic parameters in the C4 group (except panicle length) were significantly higher than those in the control (CK), with seed setting rate increasing by 8.7% (**Figures 5A–D**; **Supplementary Figures S4A, B**). Under heat stress, the C4H group (“Lifenggu”+heat) showed significantly higher SPAD values, tiller numbers, filled grains per panicle, and seed setting rate than the CKH group (heat-only control) (*P*<0.01), with seed setting rate increasing by 69% (**Figures 5A–D**; **Supplementary Table S12**). Panicle length and total grains per panicle did not differ significantly between groups (**Figures 5C, D**). Compared to their respective baselines, seed setting rate declined by 51% in C4H versus 71% in CKH, indicating significantly enhanced thermotolerance in the “Lifenggu”-treated group (**Supplementary Table S12**). Even under non-stress conditions, C4H maintained higher SPAD values and tiller numbers than CK. These results show that “Lifenggu” application enhances thermotolerance and alleviates yield losses caused by high-temperature stress.

To clarify how “Lifenggu” improves rice thermotolerance, six stress indicators (including peroxidase (POD) activity, malondialdehyde (MDA) content, hydrogen peroxide (H_2_O_2_) content, catalase (CAT) activity, superoxide dismutase (SOD) activity, and proline (Pro) content) were measured in functional leaves under high-temperature conditions (42 °C), with or without “Lifenggu” pretreatment (2.5 mL/L), and under normal conditions, with or without “Lifenggu” pretreatment (2.5 mL/L). Data analysis showed that under normal conditions, the antioxidant enzyme activities (peroxidase, superoxide dismutase, catalase), as well as proline, malondialdehyde, and hydrogen peroxide content in the C4 treatment showed no significant differences compared to CK **Figures 5E–J**; **Supplementary Table S13**). Under high-temperature stress, antioxidant enzyme activities (POD, CAT, and SOD) in C4H were significantly higher than in CKH, with increases of 28%, 11%, and 29%, respectively (**Figures 5E–G**; **Supplementary Table S13**). While the antioxidant enzyme activities in CKH decreased relative to CK, the activities of POD and CAT in C4H were significantly higher than those in CK, indicating that C4H outperformed CKH (**Figures 5E–G**). Both CKH and C4H exhibited significantly elevated levels of MDA and H_2_O_2_ compared to CK; however, MDA and H_2_O_2_ contents in the “Lifenggu”+heat group (C4H) were significantly lower than in the heat-only group (CKH), decreasing by 8.1% and 21%, respectively (**Figures 5I, J**). In contrast, proline content in both CKH and C4H was significantly higher than in CK, and Pro content in C4H increased by 22% compared to CKH (**Figure 5H**). These results suggest that the improved high-temperature stress resistance and mitigating yield losses observed in “Lifenggu”-treated rice plants is associated with modulated antioxidant enzyme activities, osmolyte accumulation, and reducing reactive oxygen species (ROS) accumulation (MDA and H_2_O_2_). This correlation may contribute to a reduction in ROS-induced damage and help maintain cellular homeostasis under high-temperature stress.

### 3.6 Transcriptome analysis

To investigate the mechanisms underlying the enhancement of grain yield and thermotolerance by “Lifenggu” application at the booting stage, this study selected the most effective concentration (C4, 2.5 mL/L) for treatment. Under normal conditions, functional leaf samples were collected 24 hours after spraying “Lifenggu” (LFG) or water (control, CK). For high-temperature stress experiments, samples were first treated with “Lifenggu” or water for 24 hours, then exposed to 42 °C for 12 hours, with samples designated as LHS (“Lifenggu”+heat stress) and HTS (heat stress-only), respectively. Transcriptome sequencing was performed on these four sample groups (CK, LFG, HTS, LHS) to analyze gene expression profiles.

**Figure 6:**
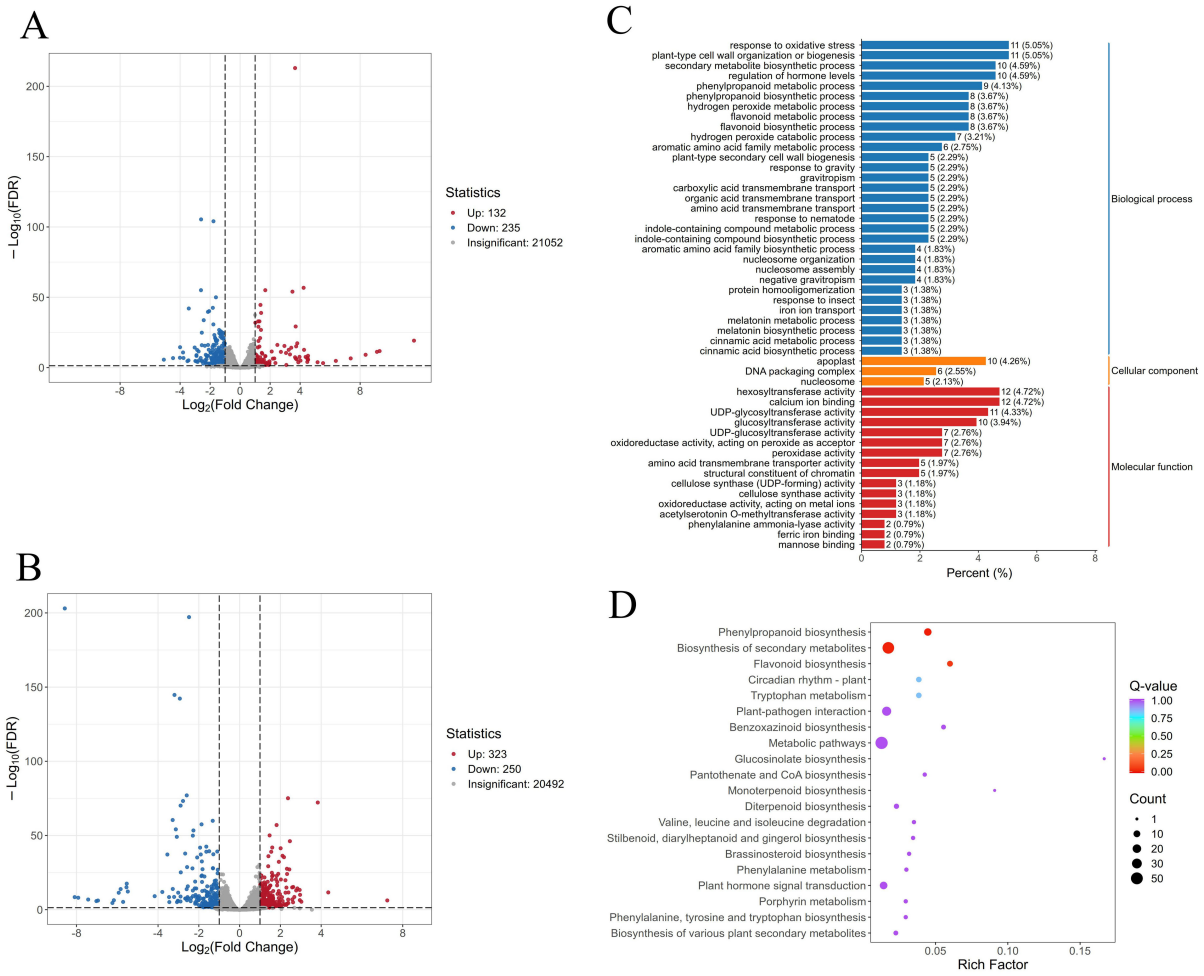
Comparative analysis of differentially expressed genes (DEGs) following “Lifenggu” application under normal and heat stress conditions. **(A)** Volcano plot of DEGs between the “Lifenggu”-treated (LFG) and control (CK) groups under normal conditions. **(B)** Volcano plot of DEGs between the “Lifenggu” + heat stress (LHS) and heat stress-only (HTS) groups. In panels **(A, B)**, red and blue points represent significantly upregulated and downregulated genes (*P* < 0.01), respectively; gray points indicate non-significant differences. **(C)** GO functional enrichment analysis of DEGs in the LFG vs. CK comparison. **(D)** KEGG pathway enrichment analysis of DEGs in the LFG vs. CK comparison. Panel **(C)** is a bar chart categorizing biological processes, cellular components, and molecular functions by percentage. **(D)** KEGG pathway enrichment analysis of DEGs in the LFG vs. CK comparison. In panel D, it illustrate various biological processes along the y-axis against a rich factor on the x-axis, the size and color of bubbles represent the number of enriched genes and *p*-values, respectively.

To visualize sample similarities and differences, principal component analysis (PCA) was performed on normalized FPKM (Fragments Per Kilobase of transcript per Million mapped reads) values of all detected genes. The PCA plot showed tight clustering of three biological replicates within each group, with clear separation of samples based on treatment conditions (**Supplementary Figure S5**), indicating high reproducibility of the sequencing data. Comparative analysis identified 358 differentially expressed genes (DEGs) between the “Lifenggu”-treated (LFG) and control (CK) groups under normal conditions, including 132 upregulated and 235 downregulated genes (**Figure 6A**). Under heat stress, 573 DEGs were detected between the “Lifenggu” + heat stress (LHS) and heat stress-only (HTS) groups, comprising 323 upregulated genes (56%) and 250 downregulated genes (**Figure 6B**).

Gene Ontology (GO) and KEGG pathway analyses were performed on DEGs identified between “Lifenggu”-treated (LFG) and control (CK) groups. The top 50 GO terms (ranked by *q*-value) comprised 31 Biological Processes (BP), 16 Molecular Functions (MF), and 3 Cellular Components (CC), highlighting the dominant role of BP and MF in “Lifenggu”-mediated growth promotion (**Figure 6C**). Specifically, BP-enriched DEGs were primarily involved in oxidative stress response, secondary metabolite biosynthesis, hormone level regulation, and phenylpropanoid metabolism, these pathways critical for stress adaptation and biosynthesis of bioactive compounds. In MF, glycosyltransferase activity was the most prominent category, linked to secondary metabolite modification and cell wall synthesis. For Cellular Components, DEGs were predominantly localized to the apoplast, DNA packaging complexes, and nucleosomes, suggesting roles in extracellular signaling and chromatin regulation. KEGG pathway analysis revealed significant enrichment in phenylpropanoid biosynthesis, secondary metabolite biosynthesis, and flavonoid biosynthesis (**Figure 6D**). Phenylpropanoids and flavonoids are key for plant stress resistance and reproductive development, directly linking transcriptional changes to enhanced growth phenotypes. Collectively, these results indicate that “Lifenggu” modulates genes involved in stress resilience and growth-promoting substance metabolism, providing a molecular basis for its growth-enhancing effects.

### 3.7 Transcriptomic insights into “Lifenggu”-mediated thermotolerance in rice

**Figure 7:**
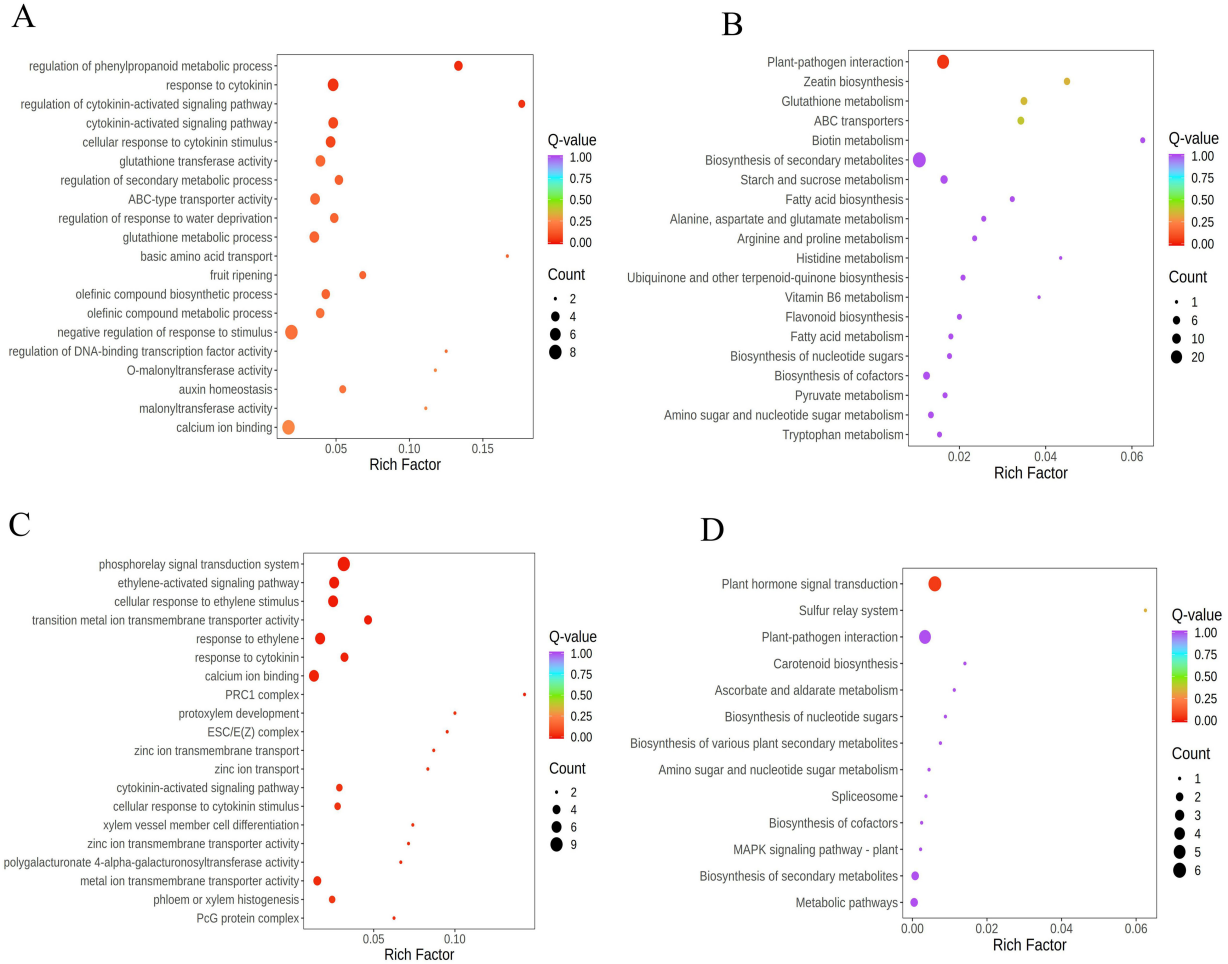
Integrated transcriptome analysis of high-temperature stress and “Lifenggu” application in rice. **(A)** GO functional enrichment analysis of differentially expressed genes (DEGs) in Class 2. **(B)** KEGG pathway enrichment analysis of DEGs in Class 2. **(C)** GO functional enrichment analysis of DEGs in Class 7. **(D)** KEGG pathway enrichment analysis of DEGs in Class 7. In all panels, they illustrate various biological processes along the y-axis against a rich factor on the x-axis. the size and color of bubbles represent the number of enriched genes and p-values, respectively.

Under stress conditions, plants dynamically adjust gene expression, with stress-responsive genes often showing significant upregulation or downregulation to enhance tolerance, mitigate damage, and sustain growth. To unravel how “Lifenggu” improves rice thermotolerance, we conducted an integrated analysis of heat stress (42 °C) and “Lifenggu” application (2.5 mL/L), focusing on Class 2 and Class 7 gene clusters (**Supplementary Figure S6**). Genes in Class 2 were upregulated under heat stress (HTS vs. CK) and further induced by “Lifenggu” (LHS vs. HTS), indicating their critical role in thermotolerance. GO enrichment analysis revealed significant clustering in phenylpropanoid metabolism regulation, cytokinin signaling, glutathione transferase activity, and secondary metabolism (**Figure 7A**), while KEGG pathways highlighted plant-pathogen interaction, zeatin biosynthesis, ABC transporters, and secondary metabolite biosynthesis (**Figure 7B**). These pathways are pivotal for synthesizing antioxidant compounds (e.g., flavonoids), modulating cytokinin-mediated stress responses, and enhancing cellular defense systems. Collectively, these findings suggest that “Lifenggu” enhances thermotolerance by upregulating genes involved in stress defense and phytohormone signaling. The enrichment of phenylpropanoid and cytokinin pathways provides a molecular basis for improved ROS scavenging and growth regulation under heat stress, establishing transcriptional reprogramming as a key mechanism for “Lifenggu”-mediated stress resilience.

In gene cluster Class 7, genes exhibited a hierarchical downregulation pattern: initial suppression under heat stress (HTS) relative to the control (CK) was further exacerbated by “Lifenggu” application (LHS). This pronounced transcriptional repression strongly suggests that the downregulation of these genes serves as a critical mechanism for enhancing rice thermotolerance. GO enrichment analysis of Class 7 DEGs revealed significant clustering in pivotal pathways, including the phosphorelay signal transduction system, ethylene-activated signaling cascades, ethylene-induced cellular responses, and cross-talk between ethylene and cytokinin signaling (**Figure 7C**). KEGG pathway analysis further highlighted enrichment in plant hormone signal transduction, plant-pathogen interaction, carotenoid biosynthesis, and multiple secondary metabolite biosynthetic pathways (**Figure 7D**). These findings indicate that “Lifenggu” modulates the expression of hormone-related genes in LHS-treated plants, potentially optimizing hormonal balance and stress adaptation to confer enhanced heat tolerance.

In addition to Class 2 and Class 7, several defense-related genes outside these clusters showed substantial upregulation in “Lifenggu”-treated samples under heat stress. Notably, the heat shock factor *LOC_Os08g43334*/*OsHsfB2b* demonstrated a 1.6-fold increase, while the cytoplasmic small heat shock protein *LOC_Os01g04370*/*OsHsp16.9A*, asparagine synthetase *LOC_Os03g18130*/*OsAS1*, and transmembrane ion transport protein *LOC_Os06g38120*/*OsLCT1* were upregulated by 1.2-, 1.2-, and 1.5-fold, respectively (**Supplementary Table S15**). These genes play pivotal roles in stress resilience: *OsHsfB2b* and *OsHsp16.9A* enhance the production of heat shock proteins to protect cellular proteins from denaturation; *OsAS1* promotes nitrogen assimilation, supporting chlorophyll biosynthesis and maintaining photosynthetic efficiency; and *OsLCT1* improves ion homeostasis and reactive oxygen species (ROS) scavenging capacity. Collectively, “Lifenggu” orchestrates a multi-faceted stress response, integrating hormonal regulation, antioxidant defense, and metabolic reprogramming to fortify rice against abiotic stress damage.

## 4 Discussion

Soil nutrient limitations pose a formidable challenge to global agricultural productivity, compelling heavy reliance on chemical fertilizers (Tilman et al., 2002). However, the indiscriminate application of these fertilizers has triggered widespread environmental degradation, including soil acidification, water eutrophication, and greenhouse gas emissions. As a result, enhancing crop nutrient use efficiency (NUE) while reducing fertilizer inputs without compromising yields has emerged as a pivotal objective in sustainable agriculture (Baligar et al., 2001). Plant biostimulants (PBs) represent a sustainable alternative, enabling the maintenance of crop productivity under reduced fertilization regimes (Gupta et al., 2020). Among these, protein hydrolysates (PHs) stand out as a promising class of PBs with significant agricultural potential. By modulating physiological processes, PHs enhance plant growth, optimize yield formation, and bolster stress tolerance, thereby offering an effective solution for promoting sustainable and resilient farming systems (Fleming et al., 2019).

Previous studies have demonstrated that plant biostimulants (PBs) can enhance growth across diverse crop species, including rice (Banakar et al., 2023; Kumar et al., 2024), wheat (Ancín et al., 2024; Mutlu-Durak et al., 2024), maize (Capo et al., 2023), lettuce (Monterisi et al., 2024), and tomato (Quintarelli et al., 2025). Notably, Colla et al. (2017a) showed that protein hydrolysates (PHs) improve chlorophyll content and physiological performance in multiple crops. In this study, application of “Lifenggu” (a protein hydrolysate) significantly increased SPAD (Soil Plant Analysis Development) values in rice leaves during both the seedling and booting stages (**Figures 2D**, **3E**). Specifically, the J treatment group (1 mL/L) induced a 97% increase in SPAD values at the seedling stage, while the C4 treatment group (2.5 mL/L) resulted in a 17% increase at the booting stage (**Supplementary Tables S6**, **S8**). Velasco-Clares et al. (2024) investigated dose-dependent biostimulant effects in lettuce and found that the optimal dosage (300 mL/hL) significantly enhanced biomass production, photosynthetic performance, and concentrations of bioactive/nutritional compounds. This treatment also activated nitrogen assimilation pathways, strengthened oxidative stress resilience via increased antioxidant levels, and improved both yield and abiotic stress tolerance. Conversely, higher doses (e.g., 500 mL/hL) risked plant mortality. Similarly, in this study, rice seedlings treated with varying PH concentrations exhibited a bell-shaped dose-response curve for seedling weight, seedling length, aboveground biomass, and SPAD values: parameters increased initially but declined with excessive PH concentrations (**Figures 2B–D**; **Supplementary Figure S1B**). Treatment J (1 mL/L) consistently yielded the most pronounced growth enhancement, establishing it as the optimal concentration. In contrast, higher-concentration treatments (L and M) significantly reduced growth parameters compared to Control A (**Supplementary Table S6**), indicating concentration-dependent growth inhibition. This study not only confirms the growth-promoting effects of PHs but also highlights the adverse impacts of excessive “Lifenggu” on rice seedlings, the findings consistent with prior reports of plant toxicity and growth suppression by commercial PHs (Cerdan et al., 2009; Lisiecka et al., 2011).

Protein hydrolysates (PHs) promote plant development by modulating key physiological and molecular pathways, with emerging evidence highlighting their role in enhancing nitrogen (N) uptake and utilization efficiency in crops (Sestili et al., 2018; Carillo et al., 2019; Choi et al., 2022). For example, Monterisi et al. (2024) demonstrated that protein hydrolysates (PHs) enhance nitrogen use efficiency (NUE) in lettuce under nitrogen-limited conditions, a mechanism linked to upregulated auxin and cytokinin biosynthesis, enhanced antioxidant capacity, and modified cell wall plasticity—synergistically driving plant growth and biomass accumulation. Similarly, Wang et al. (2022) reported that biostimulant application reduces urea fertilizer input while maintaining rice grain yield, accompanied by increased N uptake and NUE. In this study, “Lifenggu” application significantly enhanced the activities of N-metabolizing enzymes (except GLS) in the roots of rice seedlings in Treatment Group J (**Figures 2E–J**; **Supplementary Figure S1E**, **Supplementary Table S7**). During the booting stage, foliar spraying of “Lifenggu” across C1-C5 groups induced significant, dose-dependent increases in N-metabolism-related enzyme activities compared to the control (CK), with the C4 treatment (2.5 mL/L) exhibiting the most pronounced enhancement and peak enzyme activity levels (**Figures 4E–K**; **Supplementary Table S11**). Concurrently, C1-C5 treatments displayed significantly higher seed setting rates, 1000-grain weights, and grain yields per plant than CK (**Supplementary Table S10**). The strong correlation between yield improvement and N-enzyme activity trends suggests that “Lifenggu” improves grain yield through an effect on nitrogen use or efficiency. These findings reinforce the hypothesis that PHs modulate nitrogen metabolism to support sustainable crop productivity under reduced fertilization, offering a viable strategy for balancing agricultural efficiency and environmental stewardship.

Proline acts as a non-enzymatic antioxidant and is one of the most critical osmoregulators in plant cells under diverse abiotic stresses (Slabbert and Kruger, 2014). Plants with higher proline content exhibit enhanced tolerance to abiotic stressors (Hanif et al., 2021). Protein hydrolysates (PHs) have been shown to mitigate chlorophyll degradation and promote proline accumulation under stress conditions (Ertani et al., 2014). For example, Kaya (2025) demonstrated that PHs significantly improved radish (*Raphanus sativus* L.) growth under moderate salinity, evidenced by increased biomass, chlorophyll content, and enhanced developmental traits. These findings align with our herbicide-stress experiments at the seedling stage, where application of “Lifenggu” (1 mL/L) significantly enhanced SPAD values and proline content (**Figures 3E, K**). Specifically, the L+H treatment (PH + herbicide) exhibited notably higher SPAD values compared to the H treatment (herbicide alone) (**Figure 3E**). Proline concentrations in both above-ground tissues and roots were also significantly elevated in the L+H group (**Supplementary Tables S8**, **S9**). Similarly, under booting stage heat stress, the C4H treatment (2.5 mL/L “Lifenggu” + heat stress) showed significantly higher SPAD values and proline content compared to the CKH control (heat stress alone) (**Figures 5A, H**), suggesting that the enhanced stress tolerance under “Lifenggu” treatment is associated with the modulation of osmolyte accumulation. The enzymatic antioxidant system plays a pivotal role in neutralizing excess reactive oxygen species (ROS) and restoring redox balance, thereby enhancing plant adaptability to abiotic stresses (Drobek et al., 2019; Kaur et al., 2019). In the L+H group, activities of key antioxidant enzymes (POD, SOD, and CAT) were significantly higher than in the control group (**Figures 3F–H**), suggesting a potential association with enhanced herbicide stress tolerance. In our study, the pronounced increase in MDA in roots, but not in leaves, under herbicide stress may indicate a tissue-specific difference in oxidative damage or defense strategy (**Figure 3J**). This experiment was conducted using a hydroponic method. Roots, as the initial point of contact and uptake for the herbicide, might experience more severe membrane damage. Alternatively, leaves might possess a more robust or rapidly activated constitutive antioxidant system that prevents lipid peroxidation to a greater extent than in roots. Future research specifically comparing the spatiotemporal dynamics of oxidative stress across tissues would be valuable to clarify this observation. Notably, while heat stress typically suppresses antioxidant enzyme activities (Zhao et al., 2018b; Sailaja et al., 2015), the C4H treatment under heat stress exhibited significant increases in these enzymes’ activities (**Figures 5E–G**), underscoring “Lifenggu”-mediated thermotolerance. Collectively, our study provides substantial evidence supporting the potential benefits of PHs in promoting rice growth and enhancing grain yield, even under herbicide and high-temperature stress conditions. This effect is likely associated with an improvement in the plant’s physiological status, such as increased chlorophyll content, elevated proline levels, and enhanced antioxidant enzyme activities, thereby contributing to greater stress tolerance.

Cui et al. (2023) demonstrated that *Os1900* and *Os5100* play a pivotal role in regulating the conversion of carlactone (CL) to carlactonoic acid (CLA) during strigolactone biosynthesis and tiller formation in rice. Fertilizers modulate tiller number by transcriptionally regulating *Os1900*: the M4 mutant, an *Os1900* promoter deletion line, exhibited reduced *Os1900* expression, increased tillering, and higher grain yield under reduced fertilization. *LOC_Os08g41720*/*OsPIN5b* influences rice plant architecture and grain yield by regulating auxin homeostasis, transport, and distribution; reduced *OsPIN5b* expression enhances tiller number, root system vitality, panicle length, and yield (Lu et al., 2015), while its overexpression decreases cold tolerance (Fu et al., 2025). Ranjan et al. (2022) showed that knocking out LOC_Os12g12860/OsCPK29 leads to a decrease in the seed-setting rate of rice plants. Concurrently, Li et al. (2017) reported that the *LOC_Os09g25490*/*Osfc16* mutant displays growth comparable to wild-type Nipponbare but exhibits 25-41% increased biomass, reduced cellulose crystallinity, thinner secondary cell walls, and improved lodging resistance. Transcriptome analysis comparing rice treated with the protein hydrolysate “Lifenggu” (LFG) versus control (CK) revealed significant downregulation of *LOC_Os02g12890*/*Os1900*, *LOC_Os08g41720*/*OsPIN5b*, and *LOC_Os09g25490*/*Osfc16* in LFG, with fold decreases of 1.0-, 1.2-, and 2.2-fold, respectively (**Supplementary Table S15**). Correspondingly, the C4 treatment group (2.5 mL/L “Lifenggu”) exhibited a 29% increase in tillering capacity and a 22% increase in grain yield per plant compared to CK (**Supplementary Table S10**). Conversely, *LOC_Os12g12860*/*OsCPK29* (a gene critical for seed-setting rate) was upregulated by 1.23-fold in LFG versus CK (**Supplementary Table S14**), aligning with the C4 group’s 8.7% increase in seed setting rate (**Supplementary Table S10**). Collectively, these results are consistent with the notion that “Lifenggu” enhances rice yield by modulating auxin-related pathways through transcriptional regulation of tillering- and yield-associated genes (*Os1900*, *OsPIN5b*, *Osfc16*, and *OsCPK29*). The dual effects on tiller proliferation and reproductive efficiency (e.g., seed setting rate) highlight the multifaceted mechanism underlying “Lifenggu”-mediated yield improvement, establishing it as a promising tool for optimizing rice productivity through targeted gene regulation.

Heat shock factors (HSFs) and heat shock proteins (HSPs) serve as central regulators of transcriptional networks governing plant thermotolerance (Andrási et al., 2021). These molecular mediators orchestrate transcriptional reprogramming by activating downstream effector genes encoding antioxidant systems, metabolic regulators, and molecular chaperones (Nadeem et al., 2018). In this study, we observed significant upregulation of *LOC_Os01g04370*/*OsHsp16.9A* and *LOC_Os08g43334*/*OsHsfB2b* under heat stress conditions (**Supplementary Table S15**). Previous studies have established that cytosolic class I small heat shock proteins (sHSPs) in rice comprise seven members: *Hsp16.9A*, *Hsp16.9B*, *Hsp16.9C*, *Hsp17.4*, *Hsp17.7*, *Hsp17.9A*, and *Hsp18*. Compared to wild-type (WT) plants, RNA interference lines targeting class I sHSPs (RNAiCI-sHsp) exhibited delayed seed germination and impaired thermotolerance (Sarkar et al., 2020), underscoring the indispensable role of these proteins in stress adaptation. Notably, *LOC_Os08g43334*/*OsHsfB2b* has been shown to mediate drought and salt stress tolerance in rice (Xiang et al., 2013), highlighting its multifunctional role in integrating diverse abiotic stress responses. *LOC_Os03g18130*/*OsAS1*, which governs asparagine synthesis, is critical for primary NH_4_^+^ assimilation in root systems (Ohashi et al., 2015). Concurrently, Tian et al. (2019) demonstrated that rice seedlings co-expressing *OsLCT1* (*LOC_Os06g38120*), *OsHMA2*, and *OsZIP3* (termed LHZ lines) exhibited significantly higher chlorophyll content and reduced accumulation of proline, malondialdehyde (MDA), and H_2_O_2_ under cadmium (Cd) and zinc (Zn) stress compared to WT. Mechanistically, this improved stress resilience was attributed to enhanced ion transport coordination and reactive oxygen species (ROS) homeostasis, as evidenced by prolonged root and shoot growth in LHZ lines. These findings collectively highlight the interconnected roles of HSP/HSF networks and metabolic regulators in mediating plant stress tolerance.

Pathak et al. (2024) demonstrated that biostimulant application enhanced wheat yield by 40% with significant nutrient improvements, while chlorophyll content—critical for photosynthetic rate and yield—was identified as a key determinant (Gitelson et al., 2003). In the present study, a multi-location evaluation across major rice-cultivation regions in Jiangxi Province, China (encompassing varieties with diverse maturity periods) revealed that “Lifenggu” spraying significantly increased SPAD values and yield-associated parameters at all test sites, driving rice yield enhancements ranging from 8.9% to 14% (mean: 10%; **Figures 1A, B**; **Supplementary Table S5**). We speculate that the primary drivers for the yield increase are as follows: (1) shortened regreening time, which extended early tillering duration and promoted the formation of effective tillers; (2) a slower decline in SPAD values of functional leaves during the yellow ripening stage, which delayed leaf senescence, prolonged photosynthetic activity, enhanced dry matter accumulation and translocation to grains, and improved seed setting rate, 1000-grain weight, and yield per plant. Economic analysis further underscored the viability of “Lifenggu” application: the additional investment cost of 333.5 CNY/hm^-2^ for spraying was substantially lower than the increased rice output value. Based on data from 15 experimental sites and a rice purchase price of 3 CNY/kg, the average yield increase of 850.5 kg·hm^-2^ translated to an average value increase of 2551.4 CNY·hm^-2^ and a net income gain of 2217.9 CNY·hm^-2^ (**Supplementary Table S16**). Amidst global population growth and escalating sustainability imperatives, valorizing agro-industrial byproducts for protein hydrolysates (PHs) production establishes a circular bioeconomy framework. Integrating such biostimulants into crop cultivation systems not only enhances agricultural productivity but also delivers concurrent economic and ecological benefits, aligning with the principles of sustainable agriculture (van Oosten et al., 2017; Colla et al., 2015).

## 5 Conclusion

In summary, the soybean-derived protein hydrolysate (PH) “Lifenggu” exhibited a bell-shaped dose-response curve on rice seedlings: low concentrations significantly enhanced SPAD values, growth parameters, and nitrogen (N)-metabolism enzyme activities, while high concentrations suppressed growth. Treatment J (1 mL/L) identified as the ideal concentration for the seedling stage. At the booting stage, Treatment C4 (2.5 mL/L) emerged as the optimal dose, significantly improving physiological indices (e.g., chlorophyll content), yield-related traits (e.g., tiller number, seed setting rate), and N-metabolism enzyme activities. Under optimal application, “Lifenggu” enhanced resistance to herbicide and heat stress. Transcriptome analysis revealed distinct molecular mechanisms: under normal conditions (CK vs. LFG), 358 differentially expressed genes (DEGs) were enriched in pathways related to growth-promoting substance biosynthesis (e.g., auxin, cytokinin) and tiller/yield regulation (Os1900, OsPIN5b). Under heat stress (HTS vs. LHS), 573 DEGs were identified, with Class 2 and Class 7 gene clusters highlighting differential expression in biotic/abiotic stress responses, cytokinin signaling, and secondary metabolite biosynthesis. Multi-location field trials across Jiangxi Province demonstrated consistent improvements: “Lifenggu” increased rice yield by 8.9-14% (mean: + 10%) and chlorophyll content (SPAD values). In conclusion, “Lifenggu” represents a promising tool for modern agriculture, offering dual benefits of enhanced crop resilience and increased yield. Its ability to modulate hormone signaling, nitrogen metabolism, and antioxidant defenses establishes foundation for its application in climate-smart rice cultivation, aligning with global priorities for sustainable food security.

## Data Availability

The data presented in the study are deposited in the National Center for Biotechnology Information (NCBI) Sequence Read Archive (SRA) database, with the BioProject accession number PRJNA1345221.
