# Supplementary material for: Elucidating the mechanism of soybean-derived protein hydrolysate in stabilizing rice yield and enhancing agronomic efficiency
Source: Front Plant Sci. 2025 Oct 22;16:1651406. doi: 10.3389/fpls.2025.1651406 (PMC12586061; doi:10.3389/fpls.2025.1651406)
Supplement: Supplementary file 1 [file DataSheet1.docx]

Supplementary Material

# Supplementary Figures and Tables

## Supplementary Figures


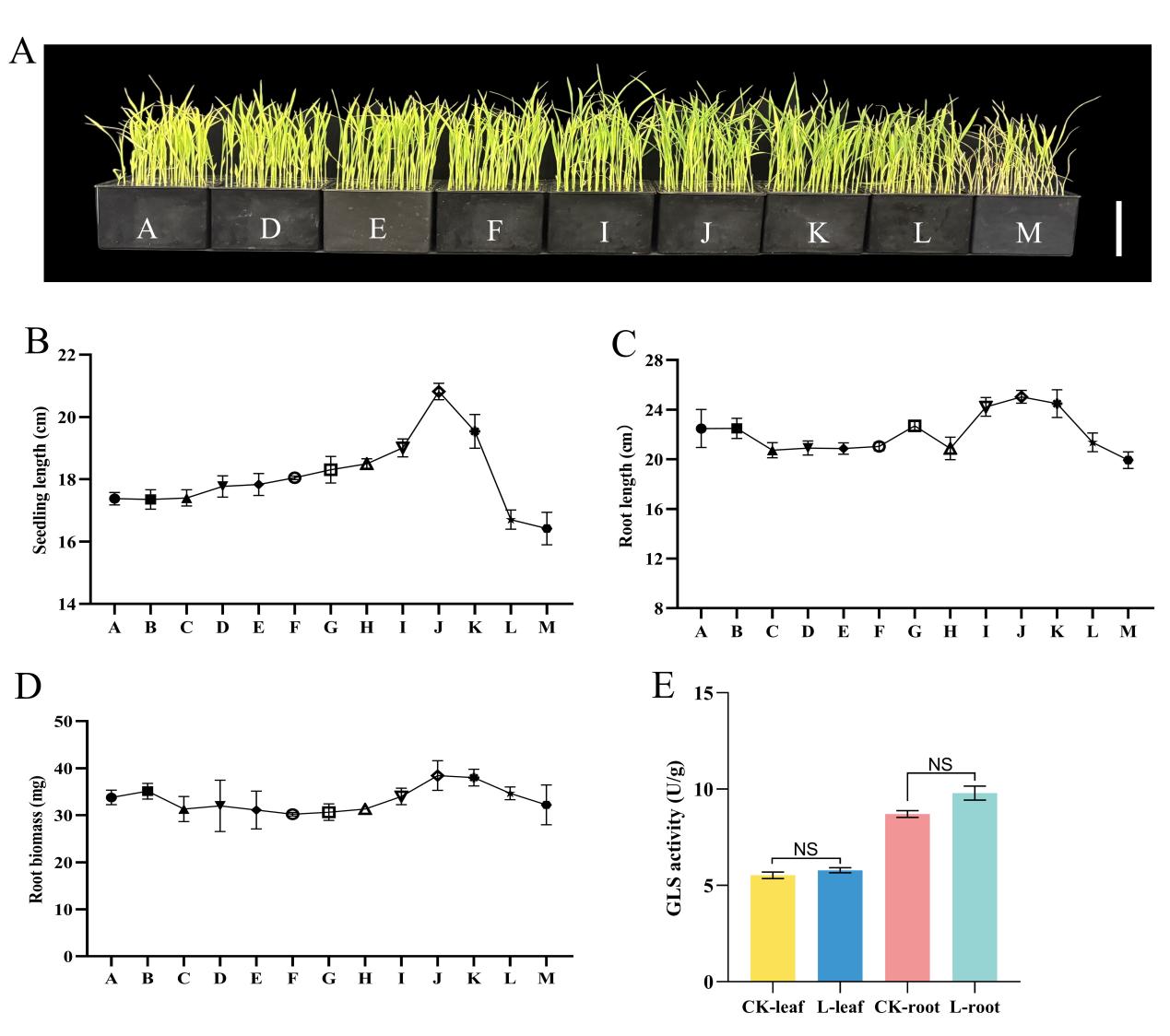


**Supplementary Figure 1.** Phenotypic and physiological analysis of rice seedlings treated with varying concentrations of "Lifenggu". (**A**) Phenotypic images of seedlings under different "Lifenggu" concentrations: A**‑**M represent treatment groups with increasing concentrations, where A is the control group. (**B‑D**) Quantitative analysis of seedling length, root length, and root biomass across treatment groups. In panels (B‑D), Groups A through M represent the experimental groups treated with different concentrations of "Lifenggu", where Group A serves as the control group. The concentrations of "Lifenggu" added are as follows: A (0 mL/L), B (0.06 mL/L), C (0.08 mL/L), D (0.13 mL/L), E (0.17 mL/L), F (0.25 mL/L), G (0.33 mL/L), H (0.50 mL/L), I (0.67 mL/L), J (1 mL/L), K (1.33 mL/L), L (2 mL/L), and M (3.33 mL/L). (**E**) Comparison of GLS (glutamine synthetase) activity in shoots and roots between the control and optimal treatment groups. NS denotes no significant difference.


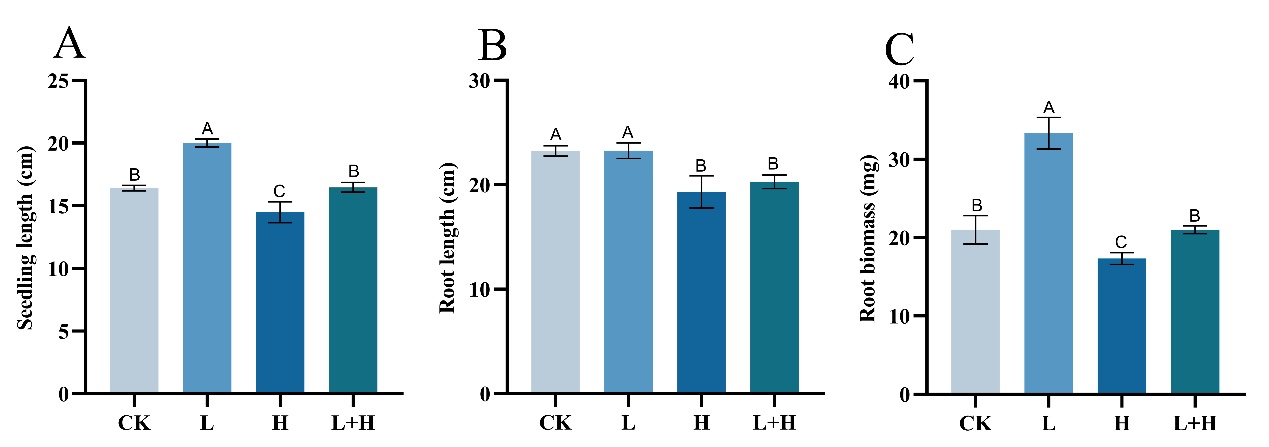


**Supplementary Figure 2.** Phenotypic analysis of rice seedlings under herbicide stress with optimal "Lifenggu" concentration at the seedling stage. In panels A-C, the concentrations of "Lifenggu" and herbicide added are as follows in four experimental groups: CK (0 mL/L+ 0 mL/L), L (1 mL/L+ 0 mL/L), H (0 mL/L+ 0.25 mL/L), L+H (1 mL/L+ 0.25 mL/L). Different uppercase letters indicate significant differences (*P* < 0.01) among treatments, while common letters denote no significant difference.


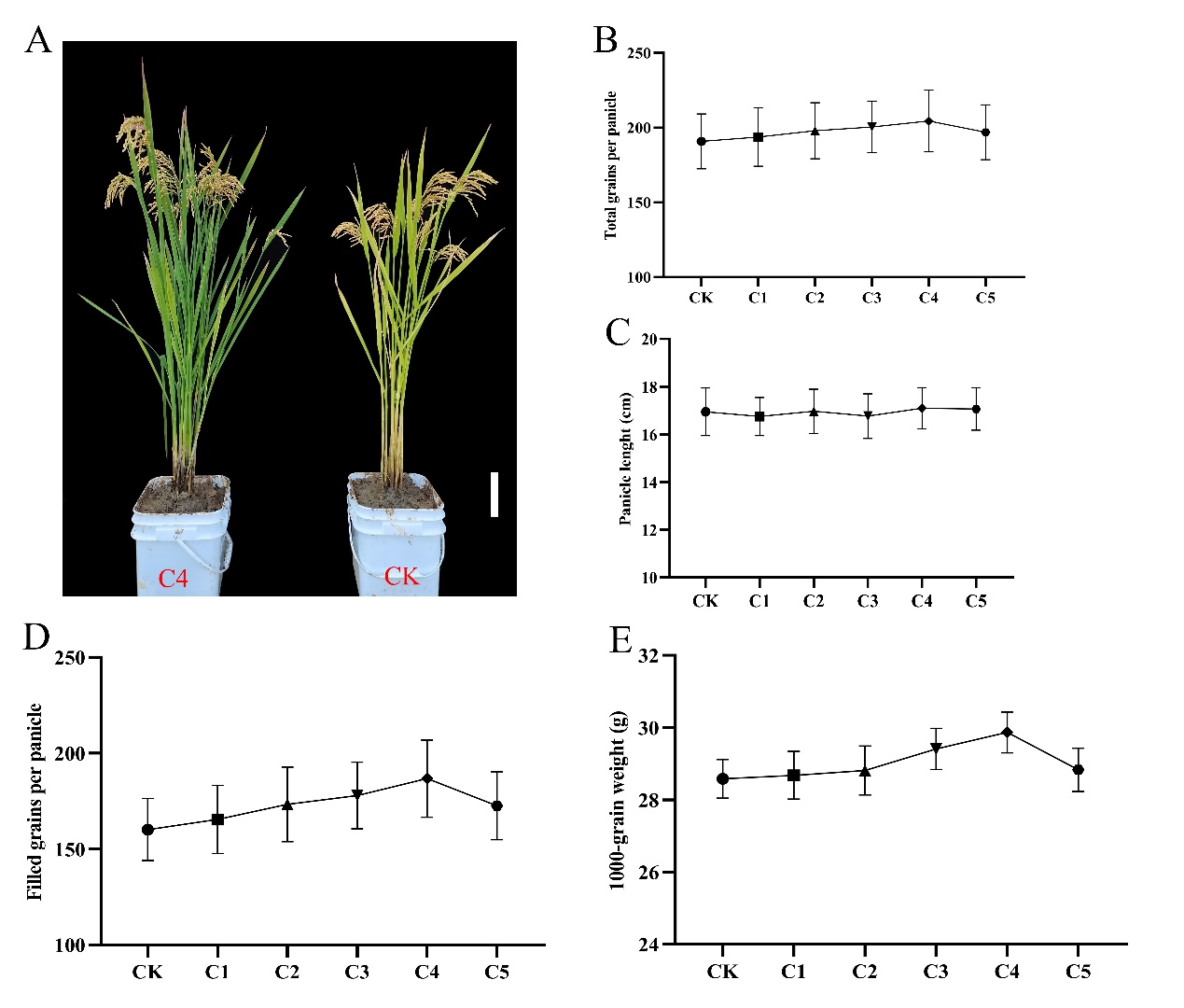


**Supplementary Figure 3.** Phenotypic analysis of rice at the booting stage under different "Lifenggu" concentrations. (**A**) Plant morphology of the C4 treatment group and control (CK), bar = 10 cm. (**B‑E**) Statistical analysis of panicle traits: total grains per panicle, filled grains per panicle, 1000-grain weight, and panicle length across C1**‑**C5 treatment groups and CK. In panels (B‑E), Groups CK and C1‑C5 represent the experimental groups treated with different concentrations of "Lifenggu", where Group CK serves as the control group. The concentrations of "Lifenggu" spraied are as follows:CK (0 mL/L), C1 (0.31 mL/L), C2 (0.67 mL/L), C3 (1.25 mL/L), C4 (2.50 mL/L), C5 (5 mL/L).


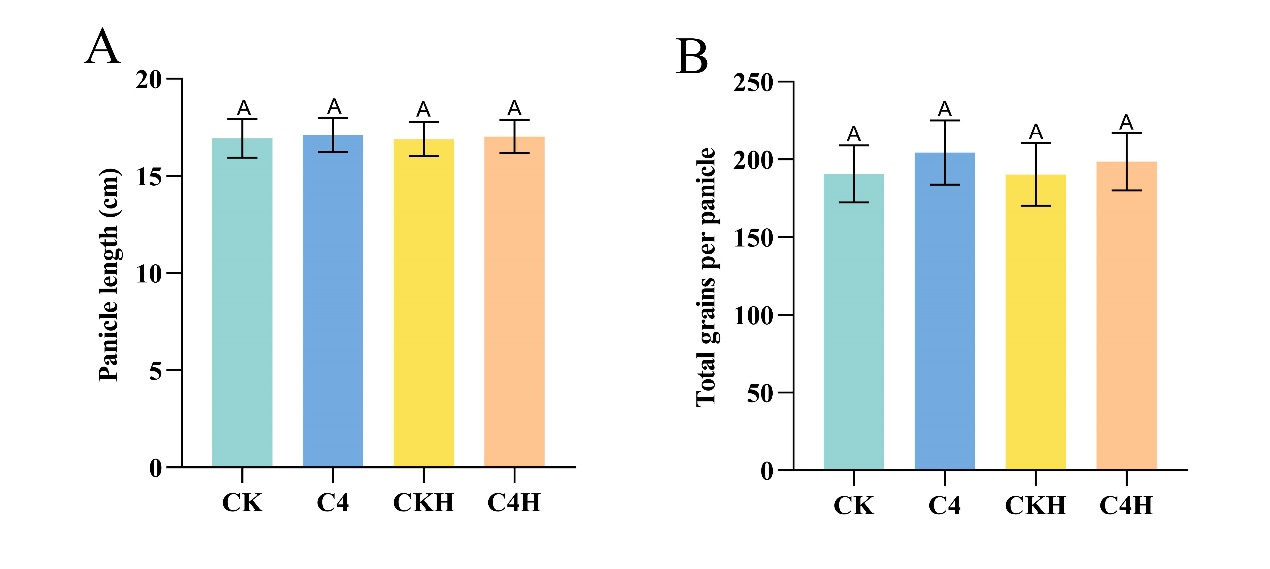


**Supplementary Figure 4.** Effects of foliar application of "Lifenggu" on high temperature stress response at the booting stage. In panels A‑B, different uppercase letters indicate significant differences (*P* < 0.01) among treatments, whereas common letters signify no significant difference.


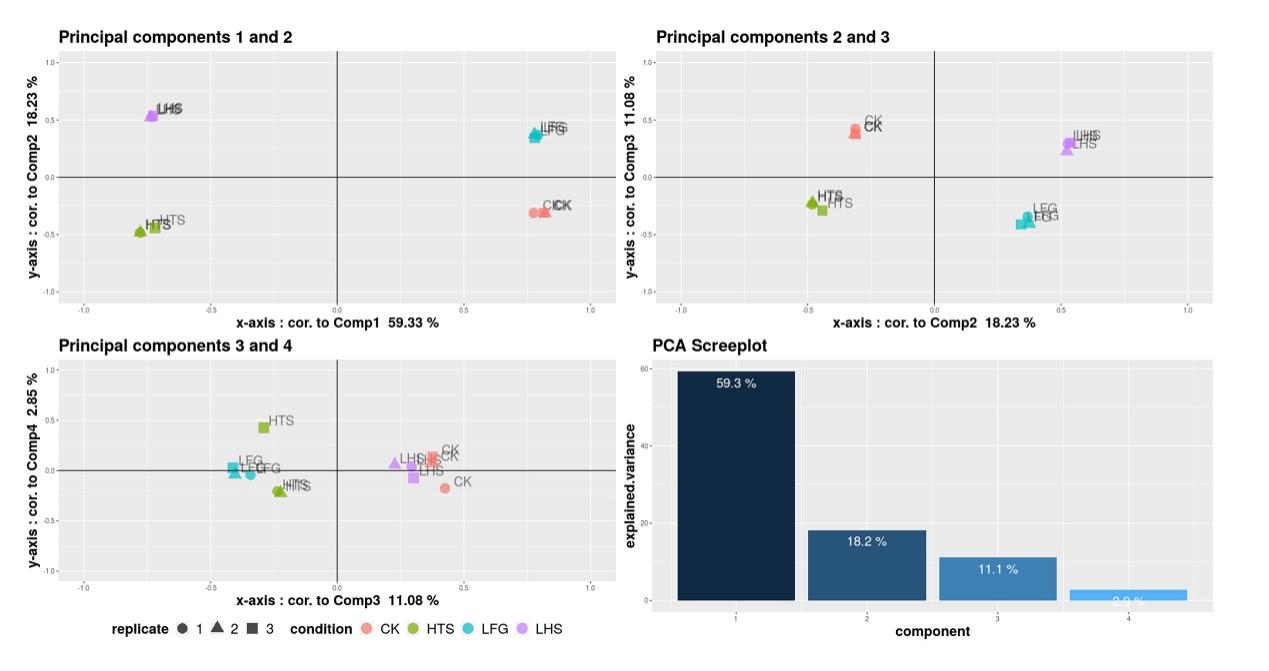


**Supplementary Figure 5.** Principal component analysis (PCA) plot.


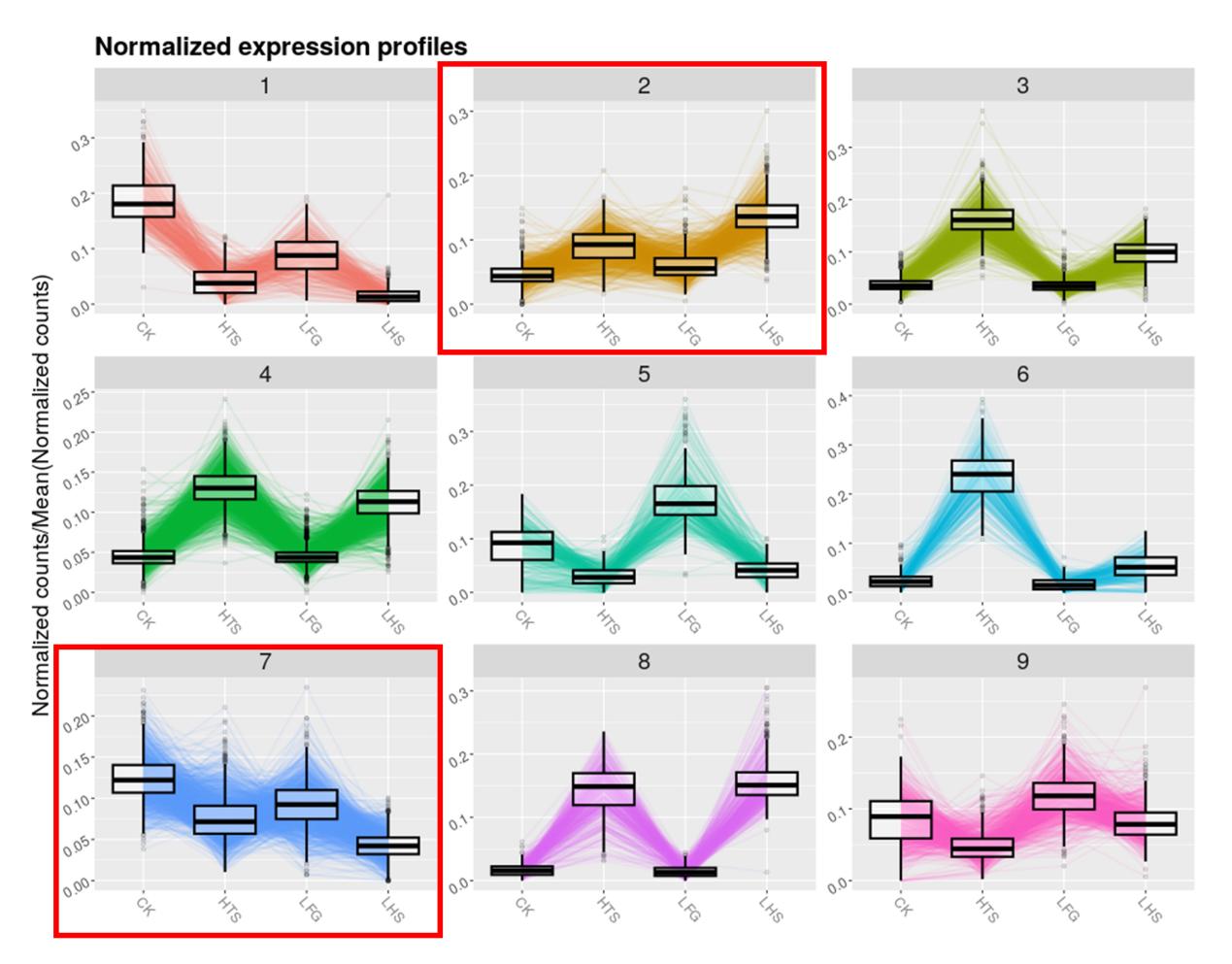


**Supplementary Figure 6.** Differential gene expression cluster analysis. Comparing gene expression profiles under normal conditions versus high-temperature stress, and "Lifenggu" application versus non-application. CK: Control group without "Lifenggu" treatment under normal conditions; HTS: Heat-stressed control group; LFG: "Lifenggu"-treated group under normal conditions; LHS: "Lifenggu"-treated group under high-temperature stress.

## Supplementary Tables

Table S1 Table of "Lifenggu" concentrations used in treatment groups of Experiment 1

| Treatment group | A | B | C | D | E | F | G | H | I | J | K | L | M |
| --- | --- | --- | --- | --- | --- | --- | --- | --- | --- | --- | --- | --- | --- |
| dilution factor | - | 16000 | 12000 | 8000 | 6000 | 4000 | 3000 | 2000 | 1500 | 1000 | 750 | 500 | 300 |
| concentration (mL/L) | - | 0.06 | 0.08 | 0.13 | 0.17 | 0.25 | 0.33 | 0.50 | 0.67 | 1.00 | 1.33 | 2.00 | 3.33 |

Table S2 Herbicide and "Lifenggu" concentrations in each treatment group of Experiment 2

| Treatment group | CK | L | H | L+H |
| --- | --- | --- | --- | --- |
| Herbicide concentration (mL/L) | 0.00 | 0.00 | 0.25 | 0.25 |
| "Lifenggu" concentration (mL/L) | 0.00 | 1.00 | 0.00 | 1.00 |

Table S3 "Lifenggu" concentrations in each treatment group of Experiment 3

| Treatment group | CK | C1 | C2 | C3 | C4 | C5 |
| --- | --- | --- | --- | --- | --- | --- |
| dilution factor | - | 2400 | 1200 | 600 | 400 | 200 |
| concentration (mL/L) | 0.00 | 0.31 | 0.63 | 1.25 | 2.50 | 5.00 |

Table S4 Basic information of the RACH pilot project

| Serial number | Experimental sites | Rice variety name | Duration |
| --- | --- | --- | --- |
| SD1 | Liantang Town, Nanchang County | Zhongzao 35 | early-season rice |
| SD2 | Xiangcheng Town, Gao 'an City | JiaYouZheKe 3 | mid-season rice |
| SD3 | Lingang Town, Leping City | Yexiang You Lisi | mid-season rice |
| SD4 | Zhongliu Village, Fengcheng City | Taoliangyou 67 | mid-season rice |
| SD5 | Huangtong Town, Jinxi County | Zhuliangyou 5298 | mid-season rice |
| SD6 | Xiangcheng Town, Gao 'an City | Demonstration material | mid-season rice |
| SD7 | Xufang Township, Jinxi County | Demonstration material | mid-season rice |
| SD8 | Quanling Miaoxia Village, Jinxian County | Huanghuazhan | mid-season rice |
| SD9 | Quanling Nan'an Village, Jinxian County | Huanghuazhan | mid-season rice |
| SD10 | Tacheng Township, Nanchang County | Conventional Glutinous Rice | mid-season rice |
| SD11 | Huangtong Town, Jinxi County | Qingxiang You 19 Xiang | late-season rice |
| SD12 | Xiangcheng Town, Gao 'an City | Fuxiang Liangyou 8 | late-season rice |
| SD13 | Xufang Township, Jinxi County | Hanliangyou 8208 | late-season rice |
| SD14 | Tacheng Township, Nanchang County | Huanghuazhan | late-season rice |
| SD15 | Liantang Town, Nanchang County | Zhongzao 35 | late-season rice |

Table S5 Statistical analysis of phenotypic data for rice yield components under "Lifenggu" spraying across different sites

| Experimental sites | Treatment group | Tiller number | Panicle length (cm) | Total grains per panicle | Filled grains per panicle | Yield per plant (g) |
| --- | --- | --- | --- | --- | --- | --- |
| SD1 | CK | 10±3 | 18.2±0.8 | 139±6 | 126±8 | 32.2±2.5 |
|  | L | 12±2*** | 18.3±0.9 | 150±8*** | 142±7*** | 36.4±4.4*** |
| SD2 | CK | 7±1 | 16.9±0.9 | 188±17 | 159±15 | 29.5±3.6 |
|  | L | 10±1*** | 17.4±0.8*** | 210±18*** | 192±17*** | 37.1±2.9*** |
| SD3 | CK | 20±3 | 24.5±1.0 | 187±15 | 140±12 | 43.7±3.5 |
|  | L | 20±3 | 24.3±0.5 | 220±22*** | 169±14*** | 48.8±5.0*** |
| SD4 | CK | 16±3 | 26.6±0.9 | 209±17 | 165±15 | 57.8±3.4 |
|  | L | 16±4 | 27.0±0.4** | 222±23*** | 179±21*** | 64.0±6.0*** |
| SD5 | CK | 14±3 | 25.3±0.8 | 195±19 | 171±16 | 50.3±4.0 |
|  | L | 16±4*** | 24.6±0.8*** | 204±26*** | 182±25*** | 57.2±6.7*** |
| SD6 | CK | 12±3 | 28.7±0.9 | 189±19 | 162±21 | 47.8±3.9 |
|  | L | 14±4 | 28.6±1.4 | 200±24*** | 180±25*** | 52.3±7.1*** |
| SD7 | CK | 15±4 | 24.3±1.2 | 187±23 | 160±21 | 48.7±3.7 |
|  | L | 17±4*** | 24.5±1.3 | 198±24*** | 181±27*** | 52.3±5.0*** |
| SD8 | CK | 5±2 | 19.9±1.1 | 124±19 | 102±19 | 10.4±3.2 |
|  | L | 5±1 | 19.2±1.3*** | 130±20*** | 116±23*** | 11.26±3.1** |
| SD9 | CK | 5±1 | 19.0±1.0 | 97±15 | 85±17 | 9.8±3.0 |
|  | L | 6±1 | 20.4±1.3*** | 109±20*** | 99±22*** | 10.6±2.8** |
| SD10 | CK | 5±1 | 19.5±1.1 | 125±18 | 106±19 | 17.8±2.6 |
|  | L | 5±2 | 19.6±1.2 | 130±22** | 119±23*** | 19.5±3.2*** |
| SD11 | CK | 13±4 | 23.6±1.3 | 165±20 | 133±22 | 34.4±3.4 |
|  | L | 15±4*** | 24.7±1.3*** | 166±26 | 136±26 | 38.6±4.6*** |
| SD12 | CK | 16±2 | 25.2±2.1 | 144±19 | 126±16 | 35.7±4.3 |
|  | L | 19±3*** | 25.5±1.1 | 154±8 | 138±8** | 39.3±6.2** |
| SD13 | CK | 14±2 | 22.8±2.0 | 160±12 | 130±11 | 31.2±4.8 |
|  | L | 15±3 | 23.1±1.2 | 172±8** | 151±8*** | 34.7±6.14** |
| SD14 | CK | 10±1 | 21.2±1.2 | 165±10 | 138±11 | 39.8±6.4 |
|  | L | 11±1 | 21.3±0.7 | 172±6** | 155±7*** | 42.1±5.4 |
| SD15 | CK | 15±2 | 24.3±1.7 | 168±10 | 150±9 | 38.7±5.8 |
|  | L | 16±2 | 24.4±1.3 | 178±6*** | 169±6*** | 40.3±4.9 |

CK represents the control group without "Lifenggu" application, while L represents the treatment group with "Lifenggu" application. ** and *** denote significant differences at the levels of *P* < 0.01 and *P* < 0.001, respectively. Data are presented as mean ± SD (t-test).

Table S6 Phenotypic statistical analysis of different concentrations of "Lifenggu" reagent applied at the seedling stage

| Treatment group | SPAD values | Seedling length (cm) | Root length (cm) | Seedling biomass (mg) | Above-ground biomass (mg) | Root biomass (mg) |
| --- | --- | --- | --- | --- | --- | --- |
| A | 19.9±3.3F | 17.4±1.2EF | 22.5±4.3CD | 115.8±12.7D | 83.8±11.8D | 34.0±8.0BC |
| B | 20.0±3.5F | 17.4±1.6EF | 22.5±4.6CD | 119.8±12.8CD | 84.7±10.5D | 35.1±6.2B |
| C | 20.2±3.8F | 17.4±1.7EF | 20.7±4.3DE | 115.6±16.7D | 84.2±15.3D | 31.3±6.2C |
| D | 21.5±3.7EF | 17.8±1.7DE | 20.9±4.7DE | 126.9±17.5CD | 94.9±14.2CD | 32.0±7.3BC |
| E | 22.88±3.9DEF | 17.84±1.8DE | 20.9±4.6DE | 126.9±19.5CD | 95.8±17.3CD | 31.1±6.4C |
| F | 23.5±4.0CDE | 18.1±1.5DE | 21.0±4.3DE | 129.6±18.7CD | 99.3±16.4C | 30.2±7.4C |
| G | 25.4±3.7CD | 18.3±1.5CD | 22.7±5.2BCD | 134.0±19.6C | 103.3±15.6C | 30.7±7.4C |
| H | 26.3±4.3C | 18.5±1.6CD | 20.9±5.2DE | 134.4±18.0C | 105.1±13.0C | 31.3±6.7C |
| I | 31.0±5.0B | 19.0±1.7BC | 24.2±4.2ABC | 158.2±16.3B | 124.2±14.6B | 34.0±6.8BC |
| J | 39.2±2.9A | 20.8±1.9A | 25.0±4.0A | 181.8±20.0A | 143.8±17.0A | 38.4±6.6A |
| K | 32.1±5.0B | 19.5±1.6B | 24.5±5.0AB | 160.7±19.2B | 122.2±17.7B | 38.00±6.2A |
| L | 23.0±2.8DEF | 16.7±1.5FG | 21.4±3.8DE | 99.3±17.1E | 64.7±16.0E | 34.7±6.3BC |
| M | 20.5±4.0EF | 16.4±1.7G | 19.9±2.6F | 95.6±14.0E | 63.3±13.8E | 32.2±6.5BC |

BM: treatment groups with different concentrations of "Lifenggu"; A: control group. Different capital letters in the figure indicate significant differences (*P* < 0.01) among treatments, while shared letters denote no significant difference. Values are presented as mean ± standard deviation (SD).

Table S7 Statistical analysis of physiological index measurements

| Physiological indicators | CK-leaf | L-leaf | CK-root | L-root |
| --- | --- | --- | --- | --- |
| GDH | 211.5±3.8 | 235.6±5.9*** | 98.4±4.1 | 121.9±3.8*** |
| GLS | 5.5±0.2 | 5.8±0.1 | 8.7±0.2 | 10.0±0.4 |
| GS | 29.0±0.2 | 28.8±0.2 | 4.0±0.1 | 7.0±0.1*** |
| NR | 1611.3±27.6 | 2507.6±31.7*** | 744.6±8.3 | 1185.6±27.6*** |
| GOGAT | 280.3±2.7 | 378.0±2.5*** | 989.6±40.0 | 1784.9±20.0*** |
| Fd-GOGAT | 2264.9±31.8 | 2726.0±25.1** | 166.8±7.7 | 309.6±17.4** |
| NiR | 3.9±0.1 | 4.2±0.1 | 1.1±0.1 | 2.3±0.2** |

CK-leaf and CK-root represent the above-ground parts and roots of control group A, respectively, while L-leaf and L-root represent the above-ground parts and roots of group J (optimal concentration treatment) after 12 hours of treatment. ** and *** denote significant differences at the levels of *P* < 0.01 and *P* < 0.001, respectively. Data are presented as mean ± SD (t-test).

Table S8 Statistical analysis of phenotypic traits in herbicide tolerance trials

| Treatment group | Seedling survival rate (%) | SPAD values | Seedling length (cm) | Root length (cm) | Seedling biomass (mg) | Above-ground biomass (mg) | Root biomass (mg) |
| --- | --- | --- | --- | --- | --- | --- | --- |
| CK | 100.0 | 23.2±3.2B | 16.4±1.1B | 23.2±4.5A | 103.5±13.5B | 82.5±13.5B | 21.0±5.8B |
| L | 100.0 | 38.5±2.1A | 20.0±1.9A | 23.3±3.6A | 150.5±15.0A | 117.2±12.6A | 33.3±6.4A |
| H | 80.3 | 17.5±1.9C | 14.5±1.9C | 19.3±4.4B | 72.0±11.9C | 54.7±9.9C | 17.3±5.5C |
| L+H | 100.0 | 22.7±3.2B | 16.5±1.3B | 20.3±3.8B | 101.0±15.3B | 80.0±13.4B | 21.0±5.5B |

Control (CK), herbicide application (H), optimal concentration of "Lifenggu" (L), and combined "Lifenggu" plus herbicide (L+H). Different capital letters in the figure indicate significant differences (*P* < 0.01) among treatments, while shared letters denote no significant difference. Data are presented as mean ± SD.

Table S9 Statistical analysis of physiological index measurement data

| Physiological indicators | H-leaf | LH-leaf | H-root | LH-root |
| --- | --- | --- | --- | --- |
| POD | 9665.0±33.9 | 10027.8±188.4 | 3292.0±27.4 | 7805.2±33.3*** |
| MDA | 9.3±0.2 | 9.8±0.1 | 5.9±0.2 | 19.2±0.5*** |
| H_2_O_2_ | 12.4±0.6 | 8.7±0.1** | 0.5±0.0 | 0.2±0.0** |
| CAT | 200.4±7.5 | 346.8±4.1* | 120.8±4.1 | 117.4±2.6 |
| SOD | 486.6±6.9 | 532.6±3.9** | 138.1±7.5 | 235.8±7.9** |
| proline | 2.6±0.0 | 2.8±0.0*** | 0.8±0.0 | 1.1±0.0*** |

"H-leaf" and "H-root" refer to the above-ground tissues and roots after 12 hours of herbicide treatment, while "LH-leaf" and "LH-root" represent the above-ground tissues and roots after 12 hours of co-treatment with "Lifenggu" and herbicide. ** and *** indicate significant differences at the *P* < 0.01 and *P* < 0.001 levels, respectively. Data are presented as mean ± SD (t-test).

Table S10 Statistical analysis of agronomic traits following spraying of different "Lifenggu" concentrations at the booting stage

| Treat-ment group | SPAD | Tiller number | Panicle length (cm) | Total grains per panicle | Filled grains per panicle | Seed setting rate (%) | 1000-grain weight (g) | Yield per plant (g) |
| --- | --- | --- | --- | --- | --- | --- | --- | --- |
| CK | 36.9±6.9C | 7±1C | 17.0±1.0A | 191±18A | 160±16BC | 84.0±3.6D | 28.6±0.5C | 30.2±3.6C |
| C1 | 38.5±6.2BC | 7±1C | 16.8±0.8A | 194±20A | 166±18BC | 85.5±3.2CD | 28.7±0.7C | 30.7±2.6C |
| C2 | 39.5±5.0ABC | 7±1C | 16.8±0.9A | 198±19A | 173±19B | 87.5±3.3BC | 28.8±0.7C | 32.4±3.1BC |
| C3 | 41.7±5.8AB | 8±1B | 16.8±0.9A | 200±17A | 178±17AB | 87.7±2.1B | 29.4±0.6B | 34.3±3.8B |
| C4 | 43.1±6.3A | 9±1A | 17.1±0.9A | 204±21A | 187±20A | 91.3±3.5A | 29.9±0.6A | 36.9±4.2A |
| C5 | 38.0±5.4BC | 7±1C | 17.1±0.9A | 197±18A | 173±18BC | 87.6±3.1B | 28.8±0.6C | 31.9±3.8BC |

CK designates the untreated control group, while C1 to C5 represent experimental groups subjected to spraying with varying concentrations of the "Lifenggu" reagent. In the figure, distinct uppercase letters signify significant differences among treatments at the *P* < 0.01 level, whereas identical letters indicate no significant difference. Data are presented as mean ± SD.

Table S11 Statistical analysis of physiological index measurement data

| Treatment group | GDH | GLS | GS | NR | GOGAT | Fd-GOGAT | NIR |
| --- | --- | --- | --- | --- | --- | --- | --- |
| CK | 171.6±3.5E | 7.8±0.1C | 15.7±0.3E | 906.8±8.0D | 92.7±4.5E | 635.3±19.7F | 3.0±0.1C |
| C1 | 193.0±2.2D | 7.5±0.1C | 19.8±0.4D | 970.1±19.7D | 125.9±4.0D | 817.0±25.1E | 3.7±0.1A |
| C2 | 196.6±5.0D | 6.9±0.1D | 24.1±0.3C | 1109.0±43.3C | 119.0±4.0D | 901.4±34.0D | 3.2±0.1BC |
| C3 | 217.0±4.1C | 8.9±0.1B | 25.4±0.3B | 1098.0±29.3C | 164.7±3.0C | 1591.0±46.3B | 3.7±0.1A |
| C4 | 256.2±4.8A | 10.1±0.2A | 30.7±0.3A | 1596.3±27.8A | 211.5±3.9A | 1784.9±29.1A | 3.4±0.1B |
| C5 | 236.0±4.6B | 5.6±0.1E | 24.9±0.2B | 1264.1±23.0B | 196.2±3.4B | 1297.7±36.0C | 3.0±0.1C |

CK represents the untreated control group, whereas C1 to C5 denote experimental groups sprayed with different concentrations of the "Lifenggu" reagent. Different uppercase letters indicate significant differences among treatments at the *P* < 0.01 level, while identical letters signify no significant difference. Data are presented as mean ± SD.

Table S12 Statistical analysis of agronomic traits of "Lifenggu" under high-temperature treatment at the booting stage

| Treatment group | SPAD | Tiller number | Panicle length (cm) | Total grains per panicle | Filled grains per panicle | Seed setting rate (%) |
| --- | --- | --- | --- | --- | --- | --- |
| CK | 36.7±6.9BC | 7±1B | 17.0±1.0A | 191±18A | 160±16B | 84.0±3.2B |
| C4 | 43.1±6.3A | 9±1A | 17.1±0.9A | 204±21A | 187±20A | 91.3±3.5A |
| CKH | 33.3±6.3C | 7±1B | 16.9±0.9A | 190±20A | 47±12D | 24.6±5.9D |
| C4H | 38.0±6.5B | 8±1A | 17.0±0.9A | 199±19A | 84±14C | 41.6±5.8C |

CK was designated as the untreated control group under normal conditions, while C4 (2.5 mL/L) represented the experimental group treated with "Lifenggu" under normal conditions. CKH served as the control group subjected to high-temperature treatment at 42°C, and C4H (2.5 mL/L) represented the experimental group simultaneously treated with high temperature and "Lifenggu". Different uppercase letters in the figure indicate significant differences (*P* < 0.01) among treatments, whereas shared letters denote no significant difference. Values are presented as mean ± SD.

Table S13 Statistical analysis of physiological index measurement data

| Physiological indicators | CK | C4 | CKH | C4H |
| --- | --- | --- | --- | --- |
| POD | 4631.6±34.9B | 4650.4±26.6B | 4179.0±44.2C | 5364.8±44.9A |
| MDA | 10.1±0.6C | 9.5±0.4C | 22.1±0.3A | 20.3±0.3B |
| H_2_O_2_ | 5.5±0.1C | 5.2±0.1C | 14.2±0.3A | 11.3±0.2B |
| CAT | 1214.3±14.6B | 1218.5±17.72B | 1184.9±24.4B | 1320.1±33.5A |
| SOD | 310.9±4.1A | 301.0±2.7A | 215.5±2.8C | 278.9±6.2B |
| proline | 1.2±0.1C | 1.1±0.0C | 2.3±0.0B | 2.8±0.0A |

CK was designated as the untreated control group under normal conditions, while C4 (2.5 mL/L) represented the experimental group treated with "Lifenggu" under normal conditions. CKH served as the control group subjected to high-temperature treatment at 42°C, and C4H (2.5 mL/L) represented the experimental group simultaneously treated with high temperature and "Lifenggu". Different uppercase letters in the figure indicate significant differences (*P* < 0.01) among treatments, whereas shared letters denote no significant difference. Values are presented as mean ± SD.

Table S14 Statistical table of selected differentially expressed genes (DEGs) in LFG versus CK

| ID | baseMean | CK | LFG | log_2_^FoldChange^ | *P* value | regulated |
| --- | --- | --- | --- | --- | --- | --- |
| LOC_Os02g12890 | 262.3 | 353.3 | 171.2 | -1.0 | 2.0×10-^9^ | down |
| LOC_Os08g41720 | 26.5 | 37.1 | 15.9 | -1.2 | 1.5×10^-3^ | down |
| LOC_Os09g25490 | 41.9 | 69.2 | 14.6 | -2.2 | 6.4×10^-11^ | down |
| LOC_Os12g12860 | 31.9 | 19.7 | 44.7 | 1.2 | 1.0×10^-3^ | up |

Table S15 Statistical table of selected differentially expressed genes (DEGs) in LHS versus HTS

| ID | baseMean | HTS | LHS | log_2_^FoldChange^ | *P* value | regulated |
| --- | --- | --- | --- | --- | --- | --- |
| LOC_Os08g43334 | 1357.2 | 672.7 | 2041.7 | 1.6 | 1.3×10^-45^ | up |
| LOC_Os01g04370 | 188.3 | 111.8 | 264.7 | 1.2 | 3.0×10^-12^ | up |
| LOC_Os03g18130 | 33.1 | 19.9 | 46.3 | 1.2 | 2.1×10^-3^ | up |
| LOC_Os06g38120 | 113.6 | 60.0 | 167.1 | 1.5 | 5.1×10-^11^ | up |

Table S16 Economic assessment of the benefits derived from the application of "Lifenggu" treatment to rice in diverse pilot projects

| Serial number | Increase production (kg·hm^-^2) | Value-added (CNY·hm^-2^) | New costs (CNY·hm^-2^) | Net increase in income (CNY·hm^-2^) |
| --- | --- | --- | --- | --- |
| SD1 | 603.0 | 1809.0 | 333.5 | 1475.6 |
| SD2 | 1076.6 | 3229.7 | 333.5 | 2896.2 |
| SD3 | 1105.5 | 3316.5 | 333.5 | 2983.1 |
| SD4 | 988.5 | 2965.5 | 333.5 | 2632.1 |
| SD5 | 805.5 | 2416.5 | 333.5 | 2083.1 |
| SD6 | 826.5 | 2479.5 | 333.5 | 2146.1 |
| SD7 | 973.5 | 2920.5 | 333.5 | 2587.1 |
| SD8 | 804.0 | 2412.0 | 333.5 | 2078.6 |
| SD9 | 798.0 | 2394.0 | 333.5 | 2060.6 |
| SD10 | 792.0 | 2376.0 | 333.5 | 2042.6 |
| SD11 | 874.5 | 2623.5 | 333.5 | 2290.1 |
| SD12 | 814.5 | 2443.5 | 333.5 | 2110.1 |
| SD13 | 768.0 | 2304.0 | 333.5 | 1970.6 |
| SD14 | 816.0 | 2448.0 | 333.5 | 2114.6 |
| SD15 | 711.0 | 2133.0 | 333.5 | 1799.6 |

The purchase price of rice is calculated at 3 CNY·kg^-1^.
